# Multi-omics analysis of the correlation between surface microbiome and metabolome in *Saccharina latissima* (Laminariales, Phaeophyceae)

**DOI:** 10.1093/femsec/fiae160

**Published:** 2025-02-21

**Authors:** Emilie Adouane, Cédric Hubas, Catherine Leblanc, Raphaël Lami, Soizic Prado

**Affiliations:** Muséum National d'Histoire Naturelle, Unité Molécules de Communication et Adaptation des Micro-Organismes, UMR 7245, CNRS, Sorbonne Université, 75005 Paris, France; Laboratoire de Biodiversité et Biotechnologie Microbienne (LBBM), Sorbonne Université, CNRS, UAR 3579, Observatoire Océanologique, 66650 Banyuls-sur-Mer, France; Muséum National d'Histoire Naturelle, Laboratoire Biologie des Organismes et Écosystème Aquatiques (UMR 8067 BOREA), Sorbonne Université, CNRS, IRD, Université de Caen Normandie, Université des Antilles, Station Marine de Concarneau, Quai de la croix, 29900 Concarneau, France; Biologie Intégrative des Modèles Marins, LBI2M (Sorbonne Université/CNRS), Station Biologique de Roscoff (SBR), 29680 Roscoff, France; Laboratoire de Biodiversité et Biotechnologie Microbienne (LBBM), Sorbonne Université, CNRS, UAR 3579, Observatoire Océanologique, 66650 Banyuls-sur-Mer, France; Muséum National d'Histoire Naturelle, Unité Molécules de Communication et Adaptation des Micro-Organismes, UMR 7245, CNRS, Sorbonne Université, 75005 Paris, France

**Keywords:** brown macroalgae, epiphytic community, holobiont, metabarcoding, metabolomics

## Abstract

The microbiome of *Saccharina latissima*, an important brown macroalgal species in Europe, significantly influences its health, fitness, and pathogen resistance. Yet, comprehensive studies on the diversity and function of microbial communities (bacteria, eukaryotes, and fungi) associated with this species are lacking. Using metabarcoding, we investigated the epimicrobiota of *S. latissima* and correlated microbial diversity with metabolomic patterns (liquid chromatography coupled to tandem mass spectrometry). Specific epibacterial and eukaryotic communities inhabit the *S. latissima* surface, alongside a core microbiota, while fungal communities show lower and more heterogeneous diversity. Metabolomic analysis revealed a large diversity of mass features, including putatively annotated fatty acids, amino derivatives, amino acids, and naphthofurans. Multiple-factor analysis linked microbial diversity with surface metabolome variations, driven mainly by fungi and bacteria. Two taxa groups were identified: one associated with bacterial consortia and the other with fungal consortia, each correlated with specific metabolites. This study demonstrated a core bacterial and eukaryotic microbiota associated with a core metabolome and highlighted interindividual variations. Annotating the surface metabolome using Natural Products databases suggested numerous metabolites potentially involved in interspecies chemical interactions. Our findings establish a link between microbial community structure and function, identifying two microbial consortia potentially involved in the chemical defense of *S. latissima*.

## Teaser

The surface of brown algae is a hub for inter-species chemical interactions.

## Introduction

Brown macroalgae, particularly the Laminariales, are important primary producers and habitat-forming species in temperate coastal ecosystems. *Saccharina latissima* (Linnaeus), CE Lane, C Mayes, Druehl, and GW Saunders, is a prominent kelp species found in European waters, playing a crucial role in nutrient provision and biodiversity maintenance (Schiel and Foster 2006, Schiel and Lilley 2007). These macroalgae form complex interactions with microbial communities, including bacteria, fungi, viruses, and archaea, collectively referred to as the algal holobiont (Wahl et al. 2012, Egan et al. 2013). Chemically mediated interactions within this holobiont influence the composition and dynamics of the algae and associated microbiota (Goecke et al. 2010, Dittami et al. 2016, Coelho-Souza et al. 2017). Two communities coexist on the alga: those living on the surface, known as epiphytes, and internal communities, called endophytes. The algal epimicrobiome is a biofilm-like microbiota known as the “second skin” (Wahl et al. 2012), which is the site of chemical and ecological interactions (Egan et al. 2013). Overall, our understanding of the diversity of colonizing microbes and their ecological roles as well as how they interact among themselves and with their hosts remains limited.

Previous studies were carried out on epimicrobiota in Laminariales species, including *Laminaria hyperborea* (Bengtsson and Øvreås 2010) and *L. digitata* (Ihua et al. 2020). However, there is limited knowledge regarding the microbiota of *S. latissima*. Studies have described the total bacterial diversity (Tourneroche et al. 2020, Burgunter-Delamare et al. 2023, Davis et al. 2023), while fungal diversity, despite its ecological significance, has been less explored. Vallet et al. (2018) emphasized the importance of endophytic fungal metabolites against protist pathogens in brown algae (Vallet et al. 2018). Although some studies have focused on endophytic fungal communities (Harvey and Goff 2010, Debbab et al. 2012, Tourneroche et al. 2020), epiphytic fungal diversity, particularly in *S. latissima*, remains understudied, with only few studies focusing on it (Chen et al. 2022, Adouane et al. 2024). To the best of our knowledge, this is the first comprehensive investigation of epiphytic fungal communities using metabarcoding.

Microorganisms in algal holobionts have strong multidirectional interactions that rely on the production of chemical signals (Goecke et al. 2010, Harder et al. 2012, Egan et al. 2013). However, our understanding of the surface-mediated signaling processes and the nature of the chemical mediators is still limited owing to methodological difficulties, such as the low concentration and water diffusion of the compounds. Metabolomics is an emerging multi-omics approach that enables the qualitative and quantitative analysis of the metabolites in a sample at a given time (Johnson et al. 2016, Parrot et al. 2019). Its application in determining the kelp surface metabolome, which can originate from both the surface microbiota and algae, can provide information on their putative interactions. Some metabolomes have already been mapped, notably that of *Fucus* spp. (Rickert et al. 2016, Parrot et al. 2019) and of *Taonia atomaria* (Othmani et al. 2016, Paix et al. 2019, 2020, 2021). Multi-omics studies, combining metabarcoding and metabolomics, on *T. atomaria* have revealed that epibacterial communities are highly specific to the algal host, correlated to specific metabolites, and that holobiont dynamics vary seasonally (Paix et al. 2019).

This study aimed to address the ecological question of links between the surface metabolome and surface microbiota, including any chemicals that may be involved in the host's defense mechanisms. To do so, multi-omics analysis was performed to characterize the epimicrobiota composition of *S. latissima* along with its surface metabolome, and to correlate variations in the metabolome with the microbial communities. This enabled us to demonstrate the presence of a core bacterial and eukaryotic microbiota associated with a core metabolome, along with interindividual variations within the algal epimicrobiota.

## Materials and methods

### Epimicrobiota sampling from *S. latissima*

Young *S. latissima* (Laminariales, Phaeophyceae) (<1 m) were harvested at Perharidy (48°43′47.0″N; 4°00′17.1″W), Roscoff, Brittany, France, in June 2021 at low tide. Six healthy (without visible damage) macroalgae were randomly selected, collected, and placed in sterile plastic bags. These samples were quickly transported to the laboratory in a cooler and then placed in a cold room (maximum 2 h) (Burgunter-Delamare et al. 2022). After rinsing each alga with sterile seawater, 5 cm high areas were delineated along the thallus of each individual (six areas in total per individual corresponding to 120 cm^2^) (Bengtsson and Øvreås 2010) (Fig. S1). These areas were extracted using two methods: one to obtain the surface microbiota and the other to obtain the surface metabolome. The surface microbiota was retrieved by scraping the algae with a scalpel and swabbing. The swabs (one per individual) were then placed in 2 ml Eppendorf tubes containing sterile seawater and vortexed for 2 min. The surface metabolome was retrieved by scraping the alga with a scalpel and placed in 2 ml of methanol (MeOH) (one sample per individual). During sampling, 2 l of seawater samples were taken and filtered on 8 µm and 0.2 µm filters each. The negative control for metabarcoding consisted of a sterile seawater sample into which a scalpel was dipped, and a swab was introduced. The sterile seawater control in which a scalpel was dipped was also used for the metabolomics method. The samples were frozen at –20 °C pending DNA extractions and mass spectrometry (MS).

### Extraction of total DNA from the algal epimicrobiota

The microbial samples (swabs and filters) were vortexed and transferred into a Lysing Matrix E tube of the FastDNA™ SPIN Kit for Soil (M.P. Biomedicals). DNA extraction was performed according to the manufacturer's instructions without any modifications. The extracted DNA was quantified using a Quantus fluorometer (Promega), and its quality was checked using a DeNovix DS-11 FX+ spectrophotometer/fluorometer.

### Identification of the microbial communities by Illumina MiSeq-based high-throughput sequencing

For amplification of the 16S and 18S rRNA genes, the prokaryotic primers U341F (5′-CCTAYGGGRBGCASCAG-3′) and U806R (5′-GGACTACNNGGGTATCTAAT-3′), and eukaryotic primers Eu565F (5′-CCAGCASCYGCGGTAATTCC-3′) and Eu981R (5′-ACTTTCGTTCTTGATYRATGA-3′) were used. Polymerase Chain Reaction (PCR) amplifications were performed (1 µl DNA-Input; 30 cycles; 1 kb ladder) according to the routine protocols of Laboratory of the Government Chemist, LGC Genomics GmbH (Berlin, Germany, http://www.lgcgroup.com, http://www.biosearchtech.com). Due to the presence of PCR inhibitors, the samples were diluted 1:10. The quality of the PCR amplifications was verified using gel electrophoresis (2% agarose). For the fungal sequences (Internal Transcribed Spacer 1, ITS1 sequences), the primers ITS1F-Kyo2 (5′-TAGAGGAAGTAAAAGTCGTAA-3′) and ITS86R (5′-TTCAAAGATTCGATGATTCAC-3′) were first used to avoid amplification of the plant and algal DNA, but no amplicon was obtained, even after dilution of the samples. The primers ITS7F (5′-GTGARTCATCGAATCTTTG-3′) and ITS4R (5′-TCCTCCGCTTATTGATATGC-3′) targeting ITS2 were used. The DNA samples were not diluted, and the quality of the PCR amplifications was checked by gel electrophoresis (2% agarose).

MiSeq paired-end sequencing (2 × 300 bp) of the 16S rRNA, 18S rRNA, and ITS2 sequences was performed using Illumina MiSeq V3 with 2.5 million reads. The resulting FASTQ files were analyzed using QIIME 2 (2022.2) (Bolyen et al. 2019) following the workflow established in the laboratory (Romani et al. 2021). Briefly, the barcodes and primers were removed by cutting adaptation (Martin 2011). The sequences were denoised, dereplicated, and matched using DADA2 (Callahan et al. 2016). Subsequently, the obtained amplicon sequence variants (ASVs) were assigned to a 99% sequence similarity with VSEARCH (Rognes et al. 2016) using the SILVA 138 database (SSU Ref. NR 138.1) (Pruesse et al. 2012, Quast et al. 2013, Yilmaz et al. 2014) for the 16S and 18S rRNA genes and the UNITE 9 database (Nilsson et al. 2019, Kõljalg et al. 2020) for ITS2. No DNA sequences from the negative control swab in sterile seawater were amplified; thus, this technical control was not included in further analysis. Subsequently, rarefaction curves were obtained, the ASVs tables were rarefied, and undesirable taxa were eliminated (mitochondria, chloroplasts, and eukaryotes for the bacterial sequences; bacteria, archaea, and vertebrates for the eukaryotic sequences; and mitochondria, chloroplasts, and other eukaryotes for the fungal sequences). All sequences were submitted to NCBI (seawater samples, accession numbers SAMN41251316–SAMN41251319 and algal epimicrobiota samples, accession numbers SAMN41249906–SAMN41249911) in BIOPROJECT PRJNA1108713.

The ASVs tables were then exported and the results were processed using Rstudio (4.2.3, 2023-03-15) with the library *phyloseq* (v.1.40.0), *ggplot2* (v.3.4.0), *vegan* (v.2.6.4), *dplyr* (v.1.0.10), *scales* (v.1.2.1), and *reshape2* (v.1.4.4) (McMurdie and Holmes 2013, 2015) on the following GitHub available workflow (https://joey711.github.io/phyloseq/). The core microbiota was identified using the *microbiome* (v1.18.0) library (Lahti and Shetty 2017) (http://microbiome.github.com/microbiome). The α-diversity indexes (Chao1, Simpson's, and Shannon) values were calculated with R, and the bar plots and statistical analyses (Mann–Whitney test) were constructed and performed, respectively, with GraphPad Prism 9.5.1 (GraphPad Software, LLC, Boston, USA).

### Metabolites extraction and liquid chromatography–mass spectrometry analysis

Samples (6) from the methanol-soaked scalpel scraping and controlled samples were filtered, evaporated, resolubilized in methanol, filtered, and dried again to remove the salt. Subsequently, they were solubilized in methanol at 10 mg.ml^−1^ and analyzed using high-performance liquid chromatography–tandem mass spectrometry (HPLC-MS/MS) in one batch and a random sequence, following previously established laboratory protocols (Vallet et al. 2018, Tourneroche et al. 2020, Adouane et al. 2024). Separation occurred onto a C18 Acclaim™ RSLC PolarAdvantage II column (2.1 × 100 mm, 2.2 μm pore size; Thermo Fisher Scientific, USA) connected to a Dionex Ultimate 3000 HPLC system and coupled to a Maxis IITM quadrupole time-of-flight–mass spectrometer (Bruker, USA) with an electrospray ionization source. The MS parameters were as follows: 3.5 kV of electrospray voltage, 35 psi of nebulizing gas (N_2_) pressure, a drying gas (N_2_) flow rate of 8 l.min^−1^, and a drying temperature of 200°C. Mobile phases comprised water (0.1% formic acid) and acetonitrile (0.1% formic acid, solvent B), following a gradient of B at 5%, 50%, 90%, and 5% for 0, 9, 15, and 21 min, respectively.

Liquid chromatography coupled to tandem mass spectrometry (LC-MS/MS) data were analyzed using DataAnalysis (version 4.4 Bruker Daltonik GmbH) and converted to “. mzXML.” The LC-MS/MS data were preprocessed on MzMine 3.2.8 with a noise level of 1E3 for MS1 and 2E1 for MS2. Further data processing was carried out according to the protocol used in our laboratory (Adouane et al. 2024). The blank was subtracted from the matrix to eliminate any compounds that may have resulted from external contamination during the experiment. The data were exported to Feature-Based Molecular Networking—Global Natural Product Social Molecular Networking (FBMN-GNPS) to build a molecular network with a cosine of 0.6 and four minimum matched fragment ions, precursor ion mass tolerance, and fragment ion mass tolerance at 0.05 Da (Wang et al. 2016, Nothias et al. 2020). Metabolites were annotated using various GNPS tools including Library search, Dereplicator, Dereplicator + (Mohimani et al. 2018) and Sirius 5.6.3 including Molecular formula identification, ZODIAC, CSI:FingerID and CANOPUS (Dührkop et al. 2015, 2019, 2021, Djoumbou Feunang et al. 2016, Ludwig et al. 2020, Kim et al. 2021). Molecular formulas were deduced from HRMS m/z values and subsequently searched and annotated using the natural products databases NPAtlas, MarinLit, and LOTUS (van Santen et al. 2019, Rutz et al. 2022, van Santen et al. 2022). Annotations were added to the network using Cytoscape 3.10.0 software. The core metabolome, representing the abundance and prevalence of redundant metabolites in the samples, was calculated from the MS data using the same R package as the metabarcoding data (see above).

### Integration of the surface metabolome and surface microbiota datasets: multi-omics approach

The LC-MS metabolomics (mass features) and metabarcoding (bacterial, eukaryotic, and fungal ASVs) data for each *S. latissima* epimicrobiota sample (epimicrobiota 1–6) were correlated. A multiple-factor analysis (MFA) was performed using Rstudio (4.2.3, 2023–03–15) with the *FactoMineR* and *factoextra* packages.

## Results

### Bacterial diversity within the epimicrobiota of *S. latissima*

The bacterial diversity of the *S. latissima* epimicrobiota was analyzed using 16S rRNA sequencing and compared with the composition of the four seawater samples. No DNA sequences were amplified from the negative control swabs in sterile seawater. The percentage of unassigned sequences in the rRNA 16S dataset was 17%–43% (Fig. S2A), corresponding to the algal mitochondrial sequences (data not shown). Percentages of abundance were calculated by excluding these sequences.

**Figure 1.**
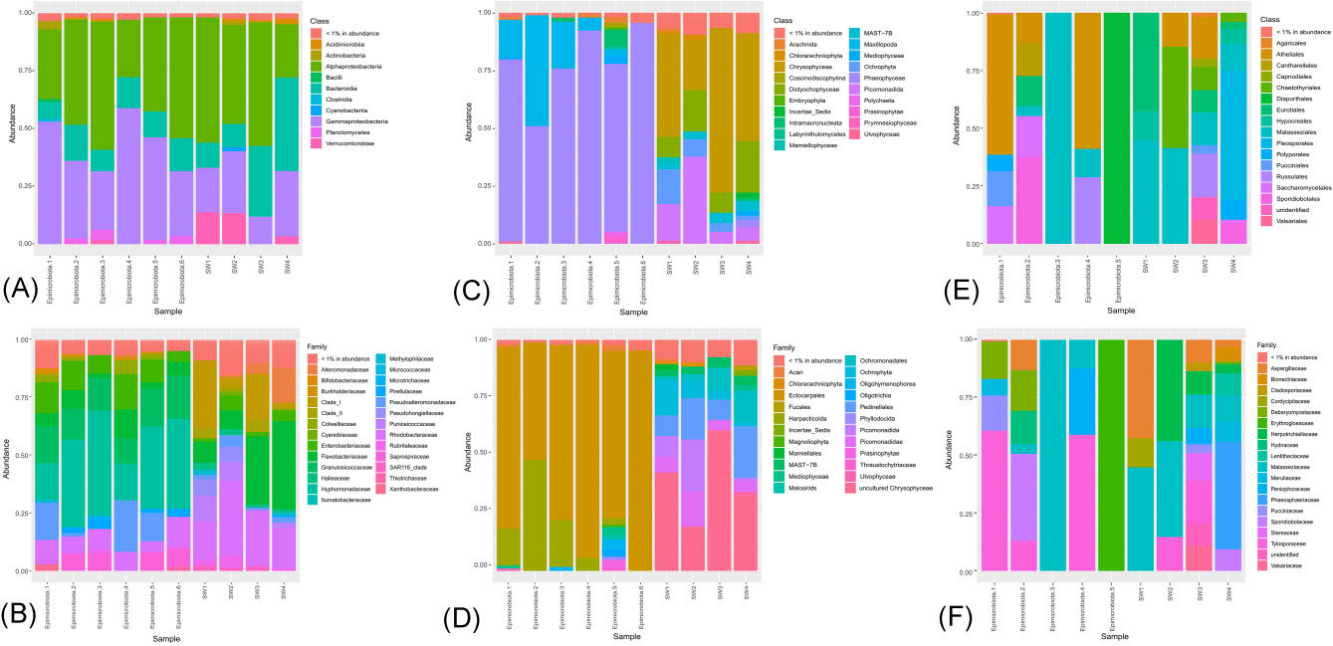
Microbial diversity within *S. latissima* epimicrobiota determined by Illumina MiSeq-based high-throughput sequencing. (A) Bacterial diversity at the class level. (B) Bacterial diversity at the family level. (C) Eukaryotic diversity at the class level. (D) Eukaryotic diversity at the family level. (E) Fungal diversity at the class level. (F) Fungal diversity at the family level. Epimicrobiota 1–6: epimicrobiota samples from *S. latissima* 1–6; SW1–SW4: seawater samples. Unassigned sequences are not shown (see Fig. S1)

Overall, high microbial diversity was observed in our samples, with 683 assigned ASVs (Table S1A). Our results showed that the algal epimicrobiota was mainly composed of Alphaproteobacteria (25%–56% of the total abundance), Gammaproteobacteria (25%–59%), and Bacteroida (7%–16%). At this taxonomic level, the seawater was composed of similar proportions of Alphaproteobacteria (23%–54%), Gammaproteobacteria (25%–59%), and 10%–40% Bacteroidetes (Fig. 1A). Within the algal epimicrobiota, the family Hyphomonadaceae was particularly present (16%–46% of total abundance) and diversified (11 ASVs), notably with *Hellea* spp., *Litorimonas* spp., and *Fretibacter* spp. (Fig. 1B and Fig. S3A). Enterobacteriaceae (*Enterobacter* spp.) (3 ASVs), Granulosicoccaceae (*Granulosicoccus* spp.) (4 ASVs), Flavobacteriaceae (41 ASVs), Saprospiraceae (*Portibacter* spp.) (9 ASVs), Rhodobacteraceae (*Sulfitobacter* spp.) (34 ASVs), and Pseudoalteromonadaceae (*Pseudoalteromonas* spp.) (3 ASVs) were also particularly abundant. In contrast, the following families were barely present in the seawater samples: Hyphomonadaceae (>1%, 7 ASVs), Enterobacteriaceae (1%–6%, 2 ASVs), Granulosicoccaceae (0%, 3 ASVs), and Pseudoalteromonadaceae (1%–5%, 3 ASVs). The seawater was mainly composed of Rhodobacteraceae (16%–32%, 56 ASVs), Flavobacteriaceae (8%–38%, 53 ASVs), and the SAR116 Clade (2%–29%, 6 ASVs) (Fig. 1B).

α-Diversity indices (Chao1, Shannon, and Simpson's) were calculated for both algal epimicrobiota and seawater samples (Fig. S4A). The Chao1 index ranged from 103 to 195 (mean 133 ± 34) for the algal epimicrobiota samples and from 178 to 300 (mean 242 ± 50) for the seawater. The Shannon index varied from 2.9 to 3.5 (mean 3.08 ± 0.17) for the algal epimicrobiota and from 3.2 to 3.9 (mean 3.57 ± 0.34) for the seawater. Calculation of the Chao1 and Shannon indices indicated that bacterial diversity in seawater was significantly higher compared to algal epimicrobiota (*P*-value = .019 and *P*-value = .038, Mann–Whitney test). However, Simpson's index revealed no significant difference in species composition between algal epimicrobiota (0.915 ± 0.009) and seawater (0.929 ± 0.023) (*P*-value = .476, Mann–Whitney test).

**Figure 2.**
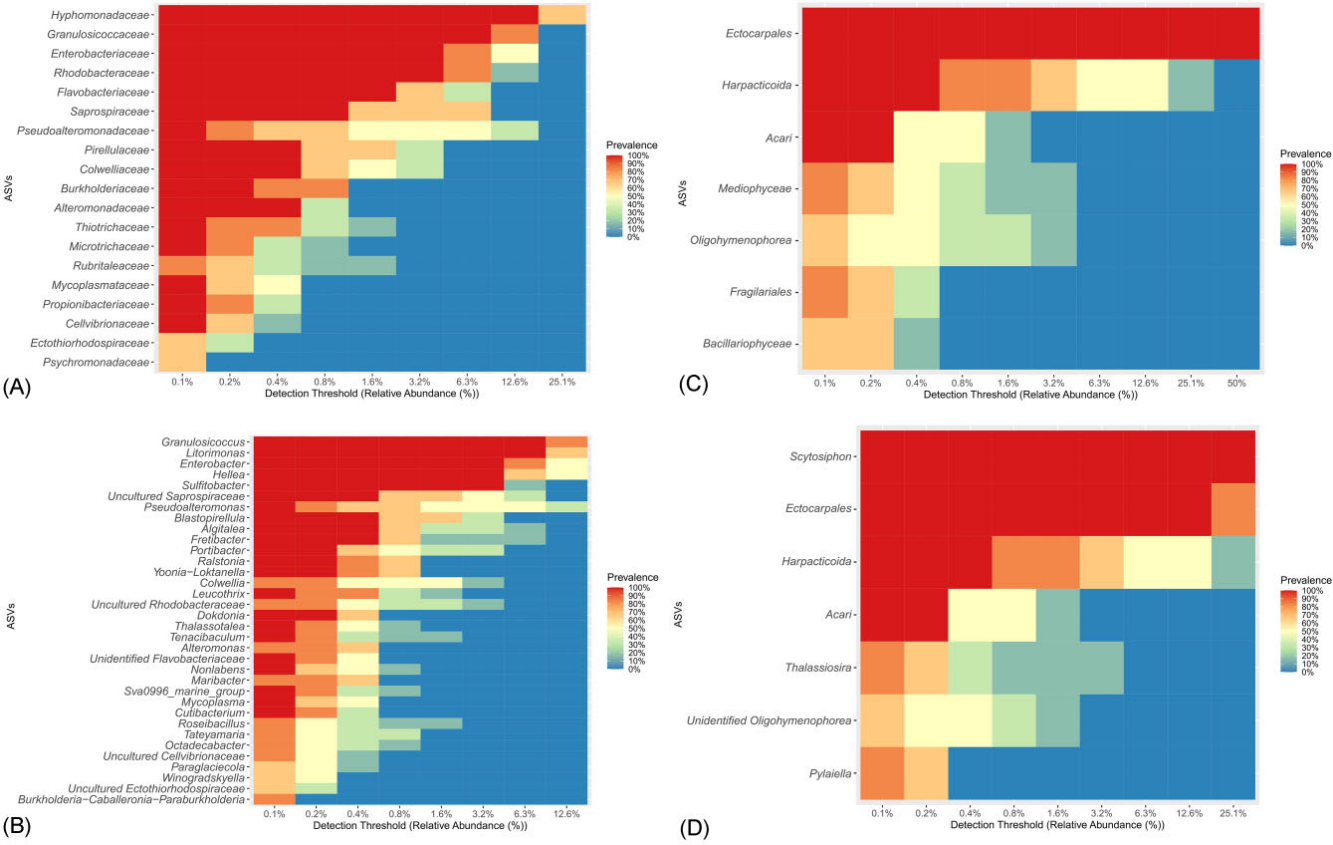
Heatmap of core microbiota within *S. latissima* epimicrobiota. (A) Bacterial core microbiota at the family level. (B) Bacterial core microbiota at the genus level. (C) Eukaryotic core microbiota at the family level. (D) Eukaryotic core microbiota at the genus level.

Subsequently, the core bacterial microbiota, which is the number of ASVs common to all the algal epimicrobiota and their relative abundances, were determined (Fig. 2A and B). The core surface microbiota of *S. latissima* comprised Hyphomonadaceae (12.6%), *Hellea* spp. (3.2%), *Litorimonas* spp. (6.3% and 0.4%), Granulosicoccaceae (6.3%), *Granulosicoccus* spp., Enterobacteriaceae (3.2%), *Enterobacter* spp., Rhodobacteraceae (3.2%), and *Sulfitobacter* spp. Thus, 25% of the surface microbiota was represented by four families and five genera.

### 18S rRNA-based analysis of eukaryotic diversity within the epimicrobiota of *S. latissima*

The eukaryotic diversity of the *S. latissima* epimicrobiota was analyzed using 18S rRNA gene sequencing. The percentage of unassigned sequences in the rRNA 18S dataset was 14%–21% for the seawater samples and 38%–91% for the algal epimicrobiota samples (Fig. S2B). Percentages of abundance were calculated by excluding these sequences. The results showed that the eukaryotic populations present in the epimicrobiota, and seawater were diverse (176 ASVs in total; Table S1B). The detected sequences were predominantly in both the epimicrobiota and seawater, affiliated with the phylum Ochrophyta (48%–96%, 30 ASVs). However, the species composition within this phylum differed between the seawater and epimicrobiota samples. The eukaryotic epimicrobiota (Fig. 1C and D) was composed of macroalgal spores belonging to the Ectocarpales family (50%–95%, 5 ASVs), especially the *Scytosiphon* spp. (27%–49%) and *Ectocarpales* spp. (8%–42%) genus but also to Arthropoda (*Harpacticoida* spp., 2 ASVs), Diatomea (*Thalassiosira* spp., 1 ASVs) and Ciliophora (*Pseudovorticella* spp., 2 ASVs) (Fig. S3B). The seawater was composed of numerous unicellular algae of the Pedinellales family (9%–22%, 2 ASVs), Ochlorophyta (*Bolidomonas* spp., 19%–60%, 4 ASVs), Ochromonadales (2%–14%, 4 ASVs), Picozoa phylum with Picomonadida (0.7%–22%, 1 ASVs), and Picomonas (5%–15%, 2 ASVs) families.

α-diversity indices (Chao1, Shannon, and Simpson's) were calculated for algal epimicrobiota and seawater samples (Fig. S4B). The Chao1 index ranged from 8 to 40 (mean 24 ± 11) for algal epimicrobiota populations and from 44 to 73 (mean 60 ± 14) for the seawater samples. The Shannon index ranged from 1.16 to 2.07 (mean 1.46 ± 0.34) for the algal epimicrobiota and from 1.77 to 2.58 (mean 2.43 ± 0.37) for seawater. The Chao1 and Shannon indices indicated that eukaryotic diversity in seawater populations is significantly higher compared to algal epimicrobiota populations (*P*-value = .0095 and *P*-value = .019, respectively, Mann–Whitney test). However, the Simpson's index revealed similar species composition between algal epimicrobiota communities (0.69 ± 0.075) and seawater (0.78 ± 0.10) (*P*-value = .257, Mann–Whitney test). Subsequently, the core eukaryotic microbiota was identified (Fig. 2C and D). The core surface eukaryotic microbiota of *S. latissima* was composed of the Ectocarpales family (50%), Scytosiphon spp. (25.1%), and Ectocarpales spp. (12.6%). Harpacticoida (0.4%) and Acari (0.2%) families were also present but in lower abundance.

### ITS-based analysis of the fungal diversity within the *S. latissima epimicrobiota*

The fungal diversity of the *S. latissima* epimicrobiota was analyzed through ITS fragment sequencing. One individual algal epimicrobiota sample (*n* = 6) was excluded from analysis due to all obtained amplicons being chimeras and sequencing errors. Between 76% and 98% of ASVs remained unassigned by the end of the analysis (Fig. S2C). These unassigned sequences mostly corresponded to abundant green algae in the Ulvaceae family. Percentages of abundance were calculated by excluding these sequences. The assignable and identifiable fungal populations (37 ASVs) (Fig. 1E and F, Table S1C) were notably lower in diversity compared to bacteria (683 ASVs) and eukaryotes (176 ASVs). At the phylum level, seawater populations comprised species mainly affiliated with Ascomycota (34%–71%, 14 ASVs) and Basidiomycota (29%–62%, 21 ASVs), while fungal epimicrobiota were predominantly affiliated with Basidiomycota (70%–100%), except for sample number 5, which was only composed of Ascomycota. The epimicrobiota sample number 5 was composed of unassigned sequences, with only one ASV remaining after eliminating these sequences, corresponding to *Dendrostoma spp*. of the Erythrogloeaceae family (Fig. 1E and F). Within the algal epimicrobiota populations (samples 1–4), the Malasseziaceae family was always present in variable proportion (1%–12%, 7 ASVs, 100% for sample number 3). Tylosporaceae (*Amphinema* spp.) were also present in the epimicrobiota samples numbered 1, 2, and 4 (13%–61%, 1 ASV). Sample number 2 was composed of the highest diversity with the presence of *Penicillium* spp. (13%, 2 ASVs), *Debaryomyces* spp. (18%, 1 ASV), *Sporobolomyces* spp. (38%, 1 ASV), and *Sistotrema* spp. (14%, 1 ASV) (Fig. S2C). However, no core metabolome has been identified. All these families were also detected in the seawater samples but included other genera, such as *Exophiala* spp., *Leptobacillium* spp., *Phaeosphaeria* spp., *Stereum* spp., and *Valsaria* spp. (1 ASV each).

The α-diversity indices (Chao1, Shannon, and Simpson's) were calculated for the fungal epimicrobiota and seawater samples (Fig. S3C). The Chao1 index ranged from 1 to 6 (mean 4.0 ± 2.0) for the fungal epimicrobiota and from 5 to 17 (mean 11.0 ± 4.6) for the seawater communities. The Shannon index was 0.85 ± 0.53 for the algal epimicrobiota and 1.82 ± 0.45 for the seawater communities. The Simpson's index was 0.443 ± 0.265 for the fungal epimicrobiota and 0.774 ± 0.079 for the seawater communities. Statistical analyses revealed no significant differences between the seawater and fungal epimicrobiota communities (*P*-values= 0.055, 0.063, and 0.111 corresponding to Chao1, Shannon, and Simpson index, respectively, Mann–Whitney U test), presenting a statistically similar number of species.

### Metabolite profile of *S. latissima* epimicrobiota

**Figure 3.**
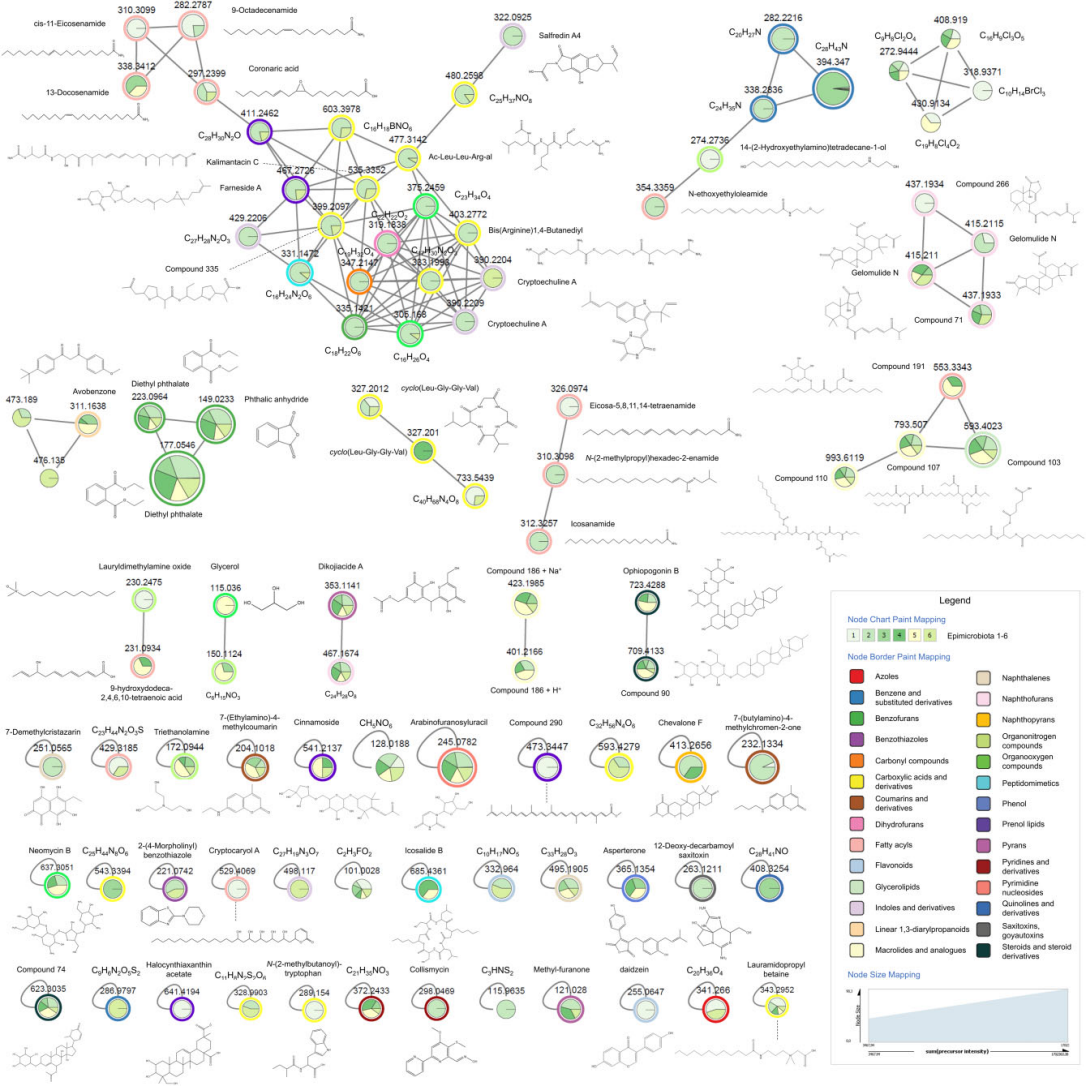
Mass features within *S. latissima* epimicrobiota. Featured-based molecular networking obtained on GNPS with parameters min pairs cos = 0.7 and minimum matched fragment Ions = 4.

The epimicrobiome of *S. latissima* was analyzed using LC-MS/MS. Following data processing, 299 mass features were detected by MS and 97 by MS/MS (Fig. S5, Tables S2 and S3). Analyses of the MS/MS data revealed seven superclasses, 28 classes, and 39 subclasses of compounds, leading to 60 putative annotations. Among the 97 mass features, seven chemical superclasses were very different, including benzenoids, lipids, lipid-like molecules, acids and their derivatives, nucleosides and their analogs, nitrogen compounds, oxygen compounds, heterocyclic compounds, phenylpropanoids, and polyketides. The predominant classes were carboxylic acids and their derivatives (15 compounds), fatty acids (12 compounds), naphthofurans (6 compounds), indoles and their derivatives (5 compounds), prenol lipids (5 compounds), macrolides (4 compounds), and flavonoids (2 compounds) as described in Fig. 3. Despite various analyses, putative annotation was difficult because many compounds were not found in the database used. However, 46 compounds were annotated as natural products produced by bacteria, fungi, plants, and diatoms (Table S2). Diethyl phthalate and phthalic anhydride were present and predominant in all the epimicrobiota. Numerous long-carbon fatty acids were annotated, including *cis*-11-eicosenamide, 13-docosenamide, 9-octadecenamide, arachidonamide, and *N*-ethoxyethyl(oleamide). Amino acid derivatives were also found, such as *cyclo*(Leu-Gly-Gly-Val) also called Leupeptin, and Kalimatacin C. Within the algal epimicrobiota, coumarins (two compounds), 7-ethylamino-4-methylcoumarin, 7-*N*-butyl-amino-4-methylcoumarin, flavonoids (two compounds) including daidzein, and compound C_10_H_17_NO_5_ were also detected.

**Figure 4.**
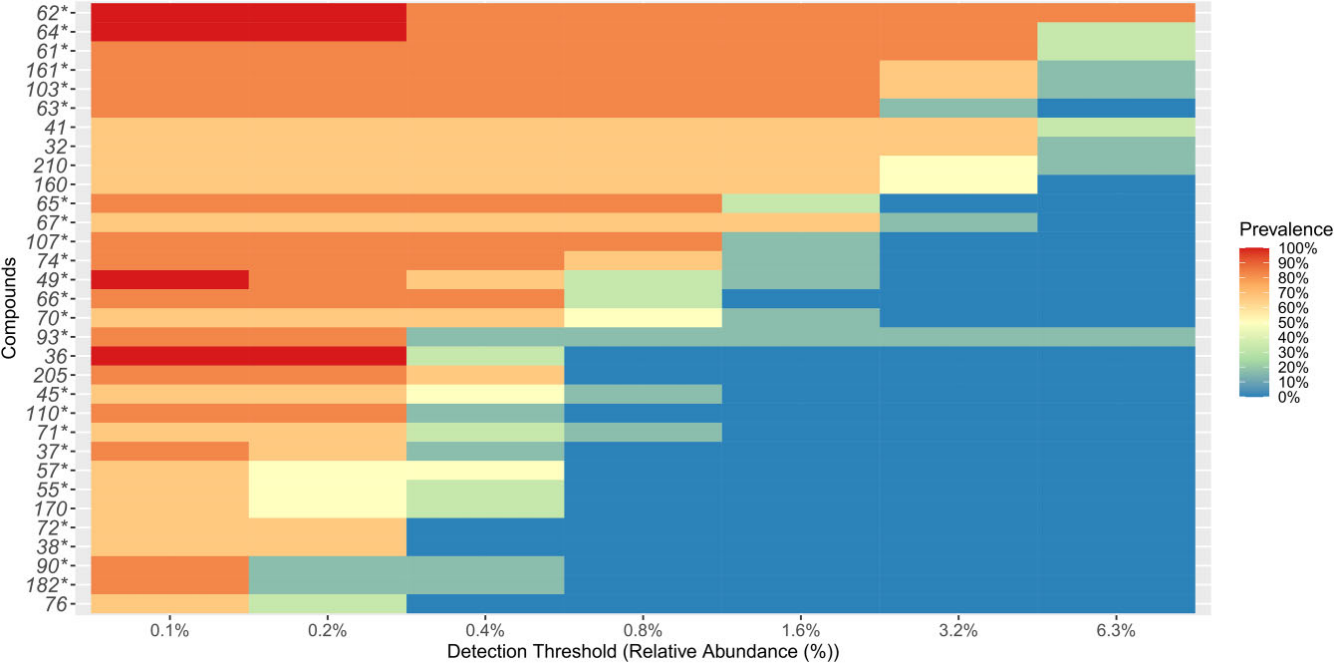
Abundance and prevalence of mass features within *S. latissima* epimicrobiota. *Compounds with a MS/MS spectra.

The core metabolome, that is, the abundance and prevalence of mass features detected by MS, was calculated, and visually represented as a heatmap (Fig. 4). The results demonstrated that the two most prevalent compounds were compounds 62 (diethyl phthalate) and 64 (arabinofuranosyluracil), which were present in 85% of the samples with an abundance of 6.3%. Compound 61, putatively identified as phthalic anhydride based on the molecular peak [M+H-C_2_H_6_O]^+^, was present in 85% of the samples, with an abundance of 3.2%. Phthalic anhydride was detected in 1.6% of the samples (Compound 63). Compounds 161 (unannotated) and 103 {5-[2,3-di(dodecanoyloxy)propoxy]-5-oxopentanoic acid, triacylglycerol (TAG)} were found in 85% of the samples, accounting for 1.6% of the total abundance. Compounds 41, 32, 210, and 160 were detected in 70% of the samples with a minimum abundance of 1.6%. However, these compounds remain unannotated as they were not fragmented during MS/MS analysis. Compounds 65 (dikojiacid A) and 107 (2,3-di(octanoyloxy)propyl 9,10,11-tri(butanoyloxy)undecanoate, macrolide lactone) were present in 90% of the samples but at a low abundance of 0.8%. In the algal epimicrobiota, compounds 49 and 36 (unfragmented) were consistently present but at very low abundances of 0.1% and 0.2%, respectively.

### Multi-omics approach to assess interindividual variation within *S. latissima* epimicrobiota

**Figure 5.**
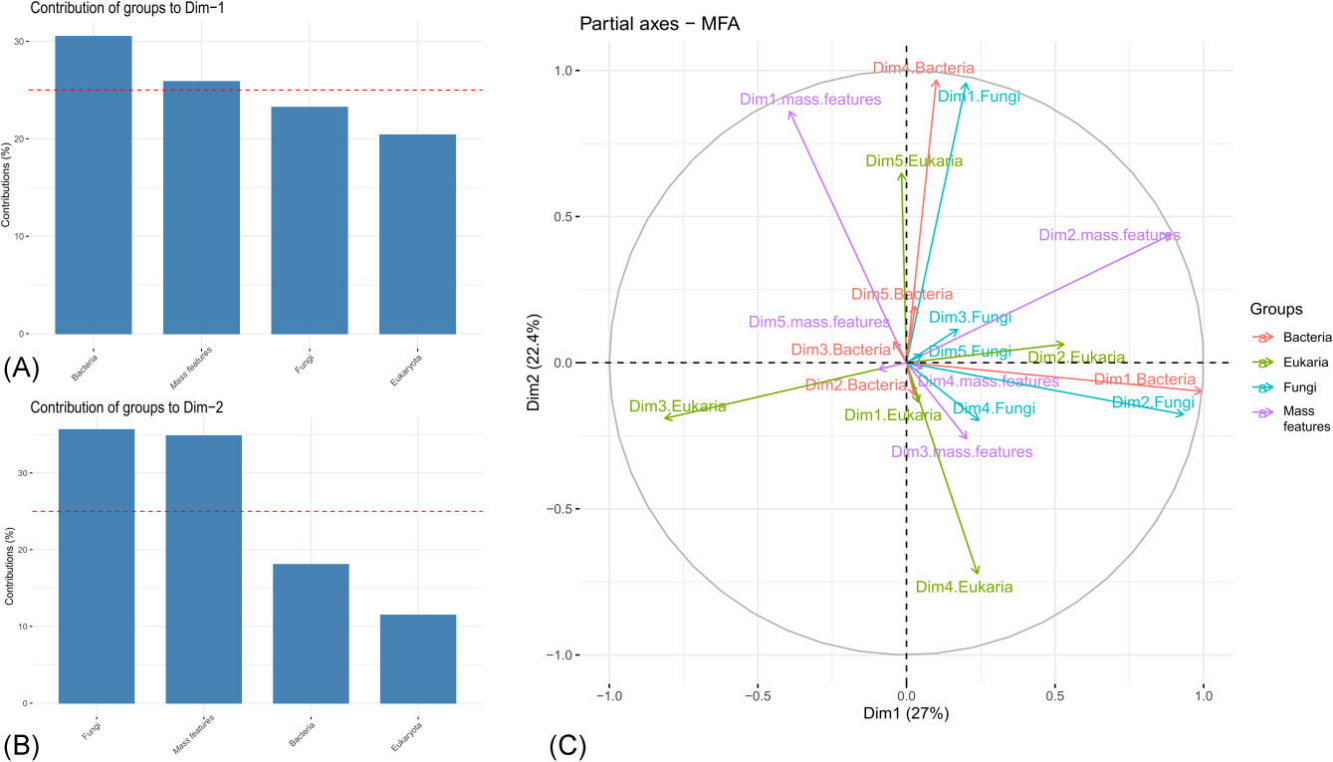
MFA result: axis representation. (A) Contribution of each group to dimension 1. (B) Contribution of each group to dimension 2. (C) Partial axes plot colored by group.

**Figure 6.**
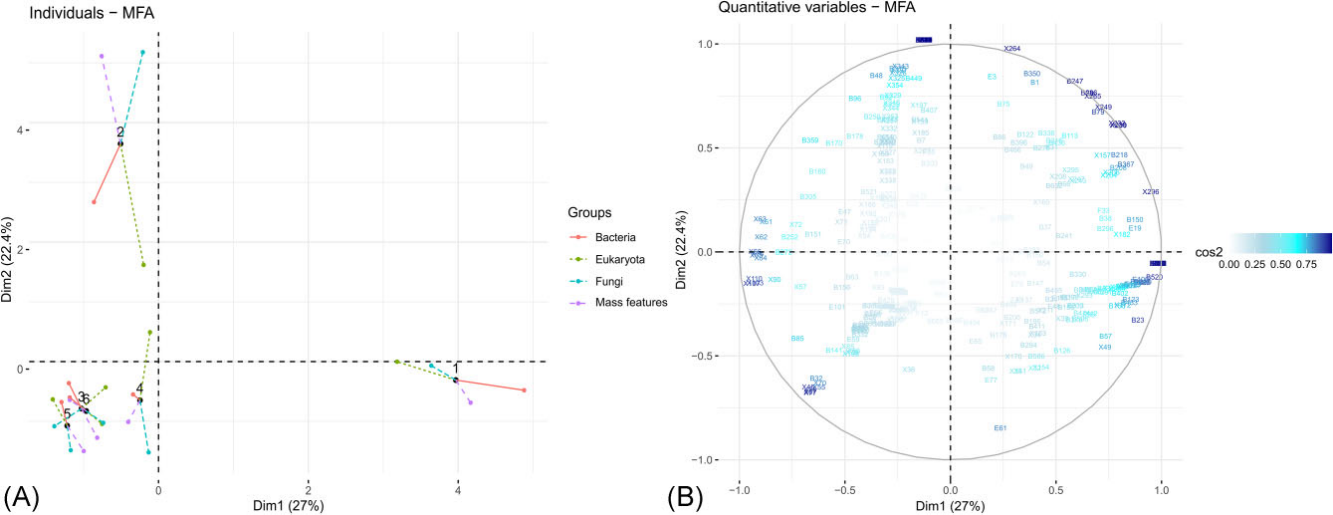
MFA result: group of variables in each individual epimicrobiota (1–6). (A) Individuals plot factor map. (B) Plot of quantitative variables colored by cos2.

The association between the microbial communities (assigned ASVs of bacteria, eukaryotes, and fungi) and mass features detected (MS data) was computed using an MFA. The graphical representation of the groups according to dimensions revealed that there was good agreement between the representation formed by the MFA and those formed by the bacteria, mass features, eukaryotes, and fungi taken separately (Fig. S6A and B). All the groups (i.e. mass features, eukaryotes, fungi, and bacteria) were relatively well represented by the first component of the MFA (bacteria: 30% contribution, mass features: 26%, eukaryotes: 20%, and fungi: 23%) (Figs 5A and  6). The bacterial group exhibited the highest correlation with the first component of the MFA (Fig. 5C, *r* = 0.95). In contrast, the second principal component of the MFA clearly separated the fungi and mass features from the bacteria and eukaryotes (Fig. S6A, fungi: 36% contribution, mass features: 26%, bacteria: 18%, and eukaryotes: 11%) (Fig. 5B and C).

An in-depth analysis of mass features or contribution of microorganisms to each dimension further highlighted the importance of fungi and eukaryotes (Fig. S6B). Indeed, because the fungal sequences were poorly represented in the dataset, they were highly structured within dimensions one and two. However, the total contribution of fungi in dimension 1 was small compared to that of the bacteria. The fungal ASVs that contributed the most to dimension 1 (Fig. S7A) were *Debaryomyces* spp. (F7), *Puccinia* spp. (F10), *Amphinema* spp. (F33), and *Phlebia* spp. (F9), and for dimension 2 were *Sistotrema* spp. (F4), *Penicillium* spp. (F18), *Sporobolomyces* spp. (F19), *Debaryomyces* spp. (F9), and *Malassezia* spp. (F35) (Fig. S7B). *Debaryomyces* spp. exhibited a strong contribution to both dimensions. However, because many microorganisms and mass features contributed to the dimensions, the significance threshold was not reached when the top 150 data points were displayed.

Variations in each group of variables for each individual epimicrobiota, that is, the epimicrobiota and their positions in relation to each other, were also studied (Fig. 6A). The axes of the partial individual factor map allowed us to observe how the individual would be placed if there was only one group of representations. The results for partial individuals showed that individuals 1 and 2 were particularly distinct from the others. Individual 1 mainly varied along dimension 1, which was well explained by all the groups, but mainly by bacteria, whereas individual 2 moved along dimension 2, which was mainly explained by a special combination of mass features and fungal sequences (Fig. 6A). Furthermore, even if the factorial axes of the MFA appeared to be represented by one group, the different variables recorded were distributed homogeneously on the MFA plane (Fig. 6B and Fig. S6B).

Focusing on the top variables (with coordinates >0.8 on dimension 1), we extracted 97 bacterial ASVs, 12 eukaryotic ASVs, and two fungal ASV covariants with 44 mass features (Table S4A) corresponding to the variability of individual 1. Among these compounds, 11 were putatively annotated, including fatty acids (arachidonamide and 9-octadecenamide), amino acids (*N*-(2-methylbutanoyl)tryptophan and lauramidopropyl betaine), isoflavones (daidzein), and triterpenoids (halocynthiaxanthin acetate). There was a wide range of bacterial ASVs, such as Proteobacteria (31), Firmicutes (24), Bacteroidota (19), Actinobacteriota (14), Acidobacteriota (1), Campilobacterota (1), Deinococcota (1), Desulfobacterota (2), Fusobacteriota (1), Myxococcota (1), Patescibacteria (1), and Verrucomicrobiota (1). The taxonomic diversity within this group of organisms was very high, with 59 bacterial families, 10 eukaryotic families, and 2 fungal families. The fungal ASVs were composed solely of Basidiomycota, *Phlebia* spp., and *Puccinia* spp. The eukaryotic ASVs were diverse, including Diatomea, Cnidaria, Porifera, Chlorophyta, Tunicata, and Basidiomycota (*Malassezia* spp.).

Focusing on the top variables with coordinates >0.8 on dimension 2, associated with individual 2, we extracted 66 mass features, as well as 36 bacterial ASVs, 3 eukaryotic ASVs, and 3 fungal ASVs (Table S4B). The bacterial ASVs included Proteobacteria (16), Bacteroidetes (11), Desulfobacteria (4), Firmicutes (1), Fusobacteria (1), Nitrospirota (1), Planctomycetota (1), and Actinobacteria (1). The eukaryotic ASVs were Diatomea (2) and Arthropoda (1), while the fungal ASVs were Ascomycota (1), Basidiomycota (2), *Penicillium* spp., *Sporobolomyces* spp., and *Sistotrema* spp. These organisms covaried with 21 partially and putatively annotated metabolites, including fatty amides such as icosanamide, icos-11-enamide, *N*-ethoxyethyl(oleamide), amino acids [bis(arginine)1,4-butanediol and Ac-Leu-Leu-Arg-al], but also a saxitoxin (12-deoxy-decarbamoylsaxitoxin), a coumarin [7-(*N*-butyl)amino-4-methylcoumarin], a naphthoquinone (7-demethylcristazarin), a benzofuranone, and a furanone (butenolides).

## Discussion

### Diversity of the microbial communities colonizing the epimicrobiota of *S. latissima* and their ecological roles

The microbial communities of *S. latissima* were identified using 16S rRNA gene sequencing to evaluate bacterial diversity, 18S rRNA gene sequencing to characterize whole eukaryotic diversity, and ITS fragment sequencing to target fungi more specifically. Results indicated distinct bacterial and eukaryotic communities specific to seawater and surface microbiota, while fungal communities on the algal surface were less diverse than in seawater and did not follow similar patterns.

Bacterial communities exhibited wide diversity in the *S. latissima* epimicrobiota, primarily comprising Alphaproteobacteria (25%–56%), Gammaproteobacteria (25%–59%), and Bacteroida, (7%–16%) consistent with microbiomes associated with brown algae (Hollants et al. 2013, Stratil et al. 2013, 2014, Parrot et al. 2019, Burgunter-Delamare et al. 2023). Our results revealed significant interindividual variations in epibacterial diversity among the six samples of *S. latissima*, consistent with prior research (Lachnit et al. 2011, Bengtsson et al. 2012, Stratil et al. 2013). These fluctuations in diversity can be attributed to various factors including temperature (Stratil et al. 2013), salinity (Stratil et al. 2014), and season (Bengtsson et al. 2010, Paix et al. 2019, Burgunter-Delamare et al. 2023), resulting in considerable variability in the microbiota composition across different types of macroalgae (Lu et al. 2023). Similar fluctuations have been observed in various parts and stages of algal growth (Staufenberger et al. 2008, Burgunter-Delamare et al. 2023).

Despite notable individual variations, our investigation revealed the presence of a core bacterial epimicrobiota. These bacterial families have been consistently identified as integral components of the core microbiota in previous studies. Notably, Granulosicoccaceae (*Granulosicoccus* spp.), has previously been observed in *Saccharina* spp. as belonging to the microbiome (Balakirev et al. 2012, Burgunter-Delamare et al. 2023); Rhodobacteraceae (*Sulfitobacter* spp.) in the *Fucus* spp. core microbiota (Stratil et al. 2013, 2014). The Hyphomonadaceae family has been depicted as part of the core microbiota of *T. atomaria* (Paix et al. 2019). These core bacterial microbiota suggest a fundamental role in the physiology of *S. latissima* as in brown alga *Fucus* spp., Rhodobacteraceae-affiliated species, notably *Sulfitobacter* spp. and *Loktanella* spp., produce vitamin B12 and siderophores that favor algal growth (Dogs et al. 2017). Previous studies have reported that some of the bacterial genera identified in the present study, including *Winogradskyella* spp. (Dong et al. 2012) and *Granulosicoccus* spp. (Weigel et al. 2022) are involved in alginate degradation, which can contribute to growth and survival by helping regenerate tissue on the surface of alginate-rich brown algae, such as *S. latissima*. Algal epimicrobiota also play a role in regulating biofouling (Holmström et al. 2002, Egan et al. 2008). For example, *Pseudoalteromonas* spp. inhibit the colonization and metamorphosis of the polychaete *Hydroides elegans* (Dobretsov and Qian 2002). This genus is also widely recognized for its antifouling activity (Egan et al. 2002, Holmström et al. 2002, Bowman 2007, Egan et al. 2008, Ballestriero et al. 2010, Dahms and Dobretsov 2017).

Unlike epibacterial communities, the diversity of fungal communities within algal holobionts remains largely unexplored and making taxonomic assignment challenging due to limited data. Recent studies have revealed that fungi establish symbiotic associations with marine macroalgae (Zuccaro et al. 2003, 2008, Harvey and Goff 2010, Loque et al. 2010, Vallet et al. 2018). However, access to the full diversity of fungal epimicrobiota was challenging since 70%–100% of the fungal sequences remained unassigned, according to the results of the few studies conducted on marine fungi. (Yao et al. 2019, Chen et al. 2022). Nevertheless, our findings revealed low fungal diversity, characterized by 37 distinct ASVs, juxtaposed with strong interindividual variability. Several families identified in our samples were also found in the endophytic microbiota of *S. latissima*, including Cladosporiaceae, Malasseziaceae, and Aspergillus (*Penicillium* spp. in our study) (Tourneroche et al. 2019, 2020). Our study revealed the presence of Hypocreales and Pleosporales, which were also present in the epimicrobiota of *Fucus* spp. (Zuccaro et al. 2003). Among the Pleosporales, *Alternaria* spp. has been detected in various macroalgae (Chen et al. 2022). Culture-dependent approaches have frequently been used for macroalgae, and the authors have managed to successfully isolate them. In this study, we detected *Penicillium* spp.*, Cladosporium* spp.*, Debaryomyces* spp.*, and Stereum* spp. which have also been isolated from culture-dependent approaches on various macroalgae (Zuccaro et al. 2003, 2008, Godinho et al. 2013, Abdel-Gawad et al. 2014, Fan et al. 2019). The functions of the fungal epimicrobiota remain poorly understood. However, it has been established that Basidiomycota likely contributes to the degradation of plant biopolymers, notably cellulose and lignin (Hyde et al. 1998, van der Wal et al. 2013). Other studies have shown that fungi such as *Mycophycias ascophylli* (order Dothideomycetes), an endophyte of *Ascophyllum nodosum*, protect algae from desiccation and produce bioactive compounds that combat pathogens (Fries 1979, 1988, Garbary and London 1995, Garbary and MacDonald 1995). In *Fucus serratus*, in contrast, spores of *Acremonium fuci* (family Hypocreales) can only germinate in the presence of algal tissue (Zuccaro et al. 2004), supporting algal–fungal interactions. In addition, certain endophytic and epiphytic fungi, notably those of the orders Pleosporales and Hypocreales, associated with brown macroalgae, also produce antibiotic, antifungal, antioxidant, and larvicidal compounds (Flewelling et al. 2015, Sarasan et al. 2017, Fan et al. 2019).

Our investigation aimed to delineate the total eukaryotic community (18S rRNA dataset) within the epimicrobiota of *S. latissima*. The results showed that the colonization of *S. latissima* was more specific than that of seawater. This eukaryotic diversity, considered as biofouling, is already a subject of interest, notably in the farm cultures of *S. latissima* (Peteiro and Freire 2013, Førde et al. 2016, Rolin et al. 2017, Matsson et al. 2019). We also showed that *S. latissima* is specifically colonized by numerous organisms (macro- and microorganisms). This diversity is mainly composed of Ectocarpales and Ochrophyta, corresponding to the macroalgal spores attached to the biofilm formed by the algal epimicrobiota, with *Ectocarpus* spp. and *Ulva* spp. frequently found in *S. latissima* cultures (Peteiro and Freire 2013). Furthermore, the abundance of macroalgal spores is influenced by their multicellular nature, resulting in a greater presence of 18S rRNA genes. The presence of Diatomea (Mediphyceae, *Thalassiosira* spp.) in the biofilm is not surprising, as they are a food source for copepods or ciliates, notably Harpacticoida and Oligomenophorea also present in the surface microbiota (Peteiro and Freire 2013).

Here, we showed that these organisms constitute the core eukaryotic epimicrobiota in *S. latissima* based on the 18S rRNA dataset. However, in literature, these organisms are typically categorized as biofouling rather than solely the “core microbiota.” Nonetheless, the coexistence of their limited abundance with the relatively small number of ASVs associated with micro- and macrofoulants, alongside the surrounding seawater, presents a complex scenario. *Saccharina latissima* seems to harbor only a few organisms, highlighting its ability to regulate biofouling. Brown macroalgae have been particularly studied for their ability to produce antibacterial compounds, including antiquorum sensing (communication mechanism based on cell density, anti-QS), antialgae, antidiatoms, and antilarvae (Saha and Wahl 2013, Chapman et al. 2014, Dahms and Dobretsov 2017, Adouane et al. 2024), but this ability has not been demonstrated for the *S. latissima* holobiont.

### Surface metabolome of *S. latissima*

We analyzed the surface metabolome of *S. latissima* using a nontargeted metabolomic approach. Annotation of metabolomes profile from marine holobionts remains challenging due to limited common databases and prior exploration, which has primarily focused on corals, sponges, and select macroalgae (Paix et al. 2019). However, putatively annotated metabolites can offer valuable insights into potential interactions among holobiont partners (Paix et al. 2019, Parrot et al. 2019).

In the surface metabolome profile of *S. latissima*, our metabolomic analyses revealed 97 mass features, 7 superclasses, 28 classes, and 39 subclasses of compounds, with 95 molecular formulas and 60 putative annotations. Fatty acids were the most common compounds, with 12 different types including cis-11-eicosenamide, 13-docosenamide, and 9-octadecenamide. While the precise role of fatty acids in macroalgal holobionts remains unclear, their diversity is significant (Paix et al. 2019, 2020). Fatty acids serve as biosurfactants and have been found across various species such as bacteria (*Bacillus* spp.), fungi, algae, plants, and sponges (Donio et al. 2013, Kendel et al. 2013, Jamal and Satheesh 2022). Hexacanamide and erucamide have been identified in several large clusters (Reddy et al. 2023). Other studies have shown that amine fatty acids are present in healthy coral microbiota and decrease during diseases (Chen et al. 2017, Pei et al. 2022). Moreover, certain fatty acids have demonstrated the ability to inhibit pathogen virulence (Xiao et al. 2004, Nobori et al. 2018). Compounds that are also widely diverse are small peptides (14) such as *N*-(2-methylbutanoyl)-tryptophan and Kalimantacin C. Low-molecular-weight peptides isolated from bacteria and fungi display general biological activities, such as antibacterial and antifungal activities (Sánchez and Vázquez 2017). Some peptides are known to inhibit QS (Gowrishankar et al. 2019, Yu et al. 2021, Wang et al. 2022, Li et al. 2023). Kalimantacin C, isolated from *Alcaligenes* spp., has strong antibiotic activity against *Staphylococcus aureus* (Kamigiri et al. 1996). In addition, we detected two naphthoquinones that could be involved in interactions between microorganisms. Quinones affect various cellular signaling pathways that promote and protect against inflammatory responses and cellular damage (Kumagai et al. 2012).

In this study, we highlight the core metabolome of the *S. latissima* epimicrobiota, composed of putatively annotated as diethyl phthlate, dikojiacid A, compound 103 {5-[2,3-di(dodecanoyloxy)propoxy]-5-oxopentanoic acid, TAG}, compound 107 [2,3-di(octanoyloxy)propyl 9,10,11-tri(butanoyloxy)undecanoate, macrolide lactone], and arabinofuranosyluracil. However, the attribution of compounds to a particular organism remains difficult because of a lack of information in the databases and the huge diversity of organisms present on the surface of *S. latissima*. Diethyl phthalate, commonly produced by bacteria and fungi like *Streptomyces* spp., *Brevibacterium* spp., and *Penicillium* spp., exhibits antimicrobial and cytotoxic activities, playing a role in various microorganism interactions (Zhang et al. 2018, Huang et al. 2021). Compound 103, a member of the TAG family, awaits further exploration regarding its function, though the presence of TAGs in macroalgae isn't unexpected due to their potential as biofuel sources. Compound 107, a macrolide lactone, lacks a defined role, yet studies suggest that similar macroalgal macrolide lactones possess antibiotic properties, produced by both bacteria and fungi (Chakraborty et al. 2020, 2021, Kizhakkepatt Kizhakkekalam et al. 2020, Carroll et al. 2022). Dikojiacid A, isolated from the plant endophyte *Aspergillus flavus*, is a natural kojic acid dimer with Cu-chelating, antiinflammatory, and antioxidant properties (Cabanes et al. 1994, Choi et al. 2012). Arabinofuranosyluracil, also known as spongouridine, has been isolated from the holobiont of the marine sponge *Tectitethya crypta* and yellow gorgonian *Eunicella cavolini* (Bergmann and Burke 1955, 1956, Cimino et al. 1984). Its production by *Vibrio harveyi* has also been demonstrated (Bertin et al. 2015). However, other epiphytic organisms such as *S. latissima* may be capable of producing it.

Among the compounds of the epimetabolome, we detected daidzein and an unknown flavonoid compound (phytoestrogen), as well as two coumarins, 7-ethylamino-4-methylcoumarin and 7-*N*-butyl-amino-4-methylcoumarin, which are potentially involved in cross-kingdom interactions. Coumarins are frequently involved in cell signaling, especially QS and biofilm formation, as activators or inhibitors (Lee et al. 2014, Gutiérrez-Barranquero et al. 2015; Coumarin: a novel player in microbial QS and biofilm formation inhibition 2018). Antibacterial, antiviral, and antifungal coumarins produced by bacteria, fungi, and algae have been reported in previous studies (Dixit et al. 2020).

Among the furanone family, which are strong QS inhibitors (Manefield et al. 2002, Kuehl et al. 2009, Phainuphong et al. 2017, Vallet et al. 2020), we detected: an unknown butenolide with the molecular formula C_22_H_22_O_2_ and a methyl-furanone. The red macroalga, *Delisea pulchra*, regulates fouling and colonization by pathogens (De Nys et al. 1995, Rasmussen et al. 2000, Harder et al. 2012). Since then, numerous furanones and butenolides produced by bacteria and fungi have shown promise as antifouling compounds (Paulitz et al. 2000, Phainuphong et al. 2017, Vallet et al. 2020). Overall, our study indicates that the *S. latissima* surface metabolome includes numerous metabolites that are potentially involved in the chemical interactions between organisms.

### Interindividual correlation of microbial diversity and metabolic profile

To draw links between the variations in the phyla and compounds, we correlated microbial community abundance and mass features abundance. We previously highlighted the existence of a bacterial microbiota core composed of abundant ASVs and a metabolome core within the epimicrobiota of *S. latissima*. The results were consistent with that of a previous study on the epiphytic bacteria of the brown alga *T. atomaria* (Paix et al. 2019, 2021). In four out of six individuals (epimicrobiome of individuals numbered 4–6), the microbial communities and associated metabolomes profiles were similar. Significant variations were observed in the microbial communities and associated mass features of the two algal samples (epimicrobiome of individuals numbered 1 and 2, Fig. 6). Unfortunately, we currently lack an explanation for these unique variations observed exclusively in two individuals. However, they give us the opportunity to study covariations between microbial communities and associated mass features.

The covariation group associated with the epimicrobiota of individual number 1 comprised bacteria (97 ASVs), eukaryotes (12 ASVs), fungi (2 ASVs), and 44 mass features (11 putatively annotated). This individual varied along dimension one, indicating that the bacteria and mass features differed significantly compared to other individuals. Here, the taxonomic diversity within this group of organisms was very high, with 59 bacterial, 10 eukaryotic, and 2 fungal families. These bacteria included Actinobacteria, Bacteroidetes, Firmicutes, and Proteobacteria. Among the eukaryotes (eukaryotic 18S rRNA-based and ITS fragment-based datasets), we found the Cercozoa, Chlorophyta, Ciliophora, Cnidaria, Diatomea, Phragmoplastophyta, Porifera, Tunicata, and Basidiomycota phyla. The metabolites putatively annotated to form this group were fatty acids (arachidonamide and 9-octadecenamide), amino acids [*N*-(2-methylbutanoyl)-tryptophan and lauramidopropyl betaine], isoflavones (daidzein), and triterpenoids (halocynthiaxanthin acetate). Moreover, fatty acid amides have been shown to have a high toxic potential for fish and grazing organisms (Bertin et al. 2015). Fucoxanthin and carotenoids, including halocynthiaxanthin acetate, are among the metabolites involved in the chemical defense of algae (Saha et al. 2011).

The second covariation group, associated with the epimicrobiota of individual number 2, comprised bacteria (36), eukaryotes (3), and fungi (3), linked to 66 mass features (21 putatively annotated). The epimicrobiota of sample number 2 was particularly interesting because it highlighted a strong covariation between the fungal communities (ITS fragment dataset) and metabolomic data, indicating that fungi may drive the production of the main chemical compounds in the epimicrobiome of this individual. This individual exhibited the highest abundance of fungi. Such observations are not surprising, as marine fungi are important producers of secondary metabolites (Hasan et al. 2015, Imhoff 2016, Deshmukh et al. 2018, Gonçalves et al. 2022). However, their diversity and roles in algal epimicrobiota remain unclear and poorly studied (Yao et al. 2019, Chen et al. 2022). This observation suggests that fungi produce specific and different metabolites depending on the algae sampled, whereas core bacteria may produce identical compounds among the different epimicrobiota. This consortium group included 20 bacterial families, as well as two eukaryotic and three fungal families. The most abundant bacterial phyla were the Bacteroidetes and Proteobacteria. The eukaryotic families (18S-based dataset) included Harpacticoida and Fragilariales and the fungal families (ITS-based dataset) included Aspergillaceae, Sporidiobolaceae, and Hydnaceae. The metabolites involved in this group were fatty amides such as icosanamide, icos-11-Enamide, *N*-Ethoxyethyloleamide, amino acids [bis(arginine)1,4-butanediol and Ac-Leu-Leu-Arg-al], but also a saxitoxin (12-deoxy-decarbamoylsaxitoxin), a coumarin [7-(*N*-butyl)amino-4-methylcoumarin], a naphthoquinone (7-demethylcristazarin), a benzofuranone and a furanone (butenolides). Within this group of covariations, defensive metabolites were also involved, including fatty acid amides (Bertin et al. 2012) and anti-QS compounds, such as furanones and their derivatives (de Nys et al. 1993, Harder et al. 2012).

The algal holobiont of *S. latissima*, like many macroalgae, is a site of intense, multidirectional communication between microorganisms (Wahl et al. 2012, Egan et al. 2013, Tourneroche et al. 2019, 2020), and our results support this view. We identified several compounds involved in the interactions between organisms, such as antibiotics (neomycin B) and phytoestrogens, and compounds involved in QS, such as coumarins. These metabolites can be produced by many organisms in response to environmental stressors or pathogen development and were not detected in individuals 1 and 2. A striking result was the covariation of furanone (butenolides, C_22_H_22_O_2_) and benzofuranone (C_18_H_22_O_6_) with several organisms, including bacteria, fungi (*Penicilliums* spp., *Sporobolomyces* spp., and *Sistotrema* spp.), and eukaryotes of the Fragilariales and Harpacticoida families. In general, QS inhibitors are produced by bacteria, fungi, and algae to regulate microbial fouling by inhibiting biofilm formation or attachment (Dobretsov et al. 2011, Stewart et al. 2014, Li et al. 2018, Yin et al. 2019, Vallet et al. 2020). Thus, fungal and bacterial communities may play a role in the defense of the algae against biofoulers and pathogens in *S. latissima*.

Overall, our analysis suggests that bacterial and fungal consortia drive the expression of a specific set of metabolites. This result is in line with previous studies on the scale of bacterial communities in algal holobionts (Burke et al. 2011b,a, Florez et al. 2017, Bonthond et al. 2020). It is also important to note that our study did not highlight the metabolic response of *S. latissima* to changes in its microbiota, which could induce the production of other compounds in response to changes in its microbial communities. Our approach enabled us to establish a link between the structure and function of microbial communities and identify two microbial consortia involved in the possible chemical defense of macroalgae.

## Supplementary Material

fiae160_Supplemental_Files
