# Supplementary material for: Multi-omics analysis of the correlation between surface microbiome and metabolome in Saccharina latissima (Laminariales, Phaeophyceae)
Source: FEMS Microbiol Ecol. 2025 Feb 21;101(3):fiae160. doi: 10.1093/femsec/fiae160 (PMC11879540; doi:10.1093/femsec/fiae160)
Supplement: fiae160_Supplemental_Files [file fiae160_supplemental_files.zip › Supplementary figures.docx]

**SUPPLEMENTARY FIGURES: TITLE AND LEGENDS**

**Supp. Fig. 1** Methods for sampling *S. latissima* epimicrobiota

**Supp. Fig. 2** Microbial diversity at the Family level with unassigned sequences within *S. latissima* epimicrobiota determined by Illumina MiSeq-based high-throughput sequencing. **(A)** Bacterial diversity (RNAr 16S gene) **(B)** Eukaryotic diversity (RNAr 18S gene) **(C)** Fungal diversity (ITS2 gene). Epimicrobiota 1-6: epimicrobiota samples from *S. latissima* 1- 6; SW1-SW4: seawater samples.

**Supp. Fig. 3** Microbial diversity at the Genus level without unassigned sequences within *S. latissima* epimicrobiota **(A)** Bacterial diversity (RNAr 16S gene) **(B)** Eukaryotic diversity (RNAr 18S gene) **(C)** Fungal diversity (ITS2 gene). Epimicrobiota 1-6: epimicrobiota samples from S. latissima 1-6; SW1-SW4: seawater samples.

**Supp. Fig. 4** Alpha diversity index of Bacterial (A), Eukaryotic diversity (B) and Fungal Diversity (C) (Mann-Whitney test, GraphPad Prism 9.5.1).

**Supp. Fig 5** Molecular network from FBMN without annotation

**Supp. Fig. 6** MFA construction dimension (A) Groups representation (B) Partial axes plot colored by group.

**Supp. Fig. 7** Contribution of top 150 quantitative variables to dimension (A) Dim-1 (B) Dim-2

**Supp. Table 1**: Composition within *S. latissima* epimicrobiota (A) Bacterial ASV (B) Fungal ASV (C) Eukaryotic ASV. Epimicrobiota 1-6: epimicrobiota samples from *S. latissima* 1-6; SW1-SW4: seawater samples.

**Supp. Table 2:** Putative annotation of mass features within *S. latissima* epimicrobiota

**Supp. Table 3:** MS and MS2 features. *S. latissima* epimicrobiota samples and quantification for each sample. Data treated on MzMine 3.2.8.

**Supp. Table 4**: Identification of clustering quantitative variables (coord > 0.8) for each dimension (A) Individual 1 (B) Individual 2
