# Supplementary figures and images for: Multi-omics analysis of the correlation between surface microbiome and metabolome in Saccharina latissima (Laminariales, Phaeophyceae)
Source: FEMS Microbiol Ecol. 2025 Feb 21;101(3):fiae160. doi: 10.1093/femsec/fiae160 (PMC11879540; doi:10.1093/femsec/fiae160)

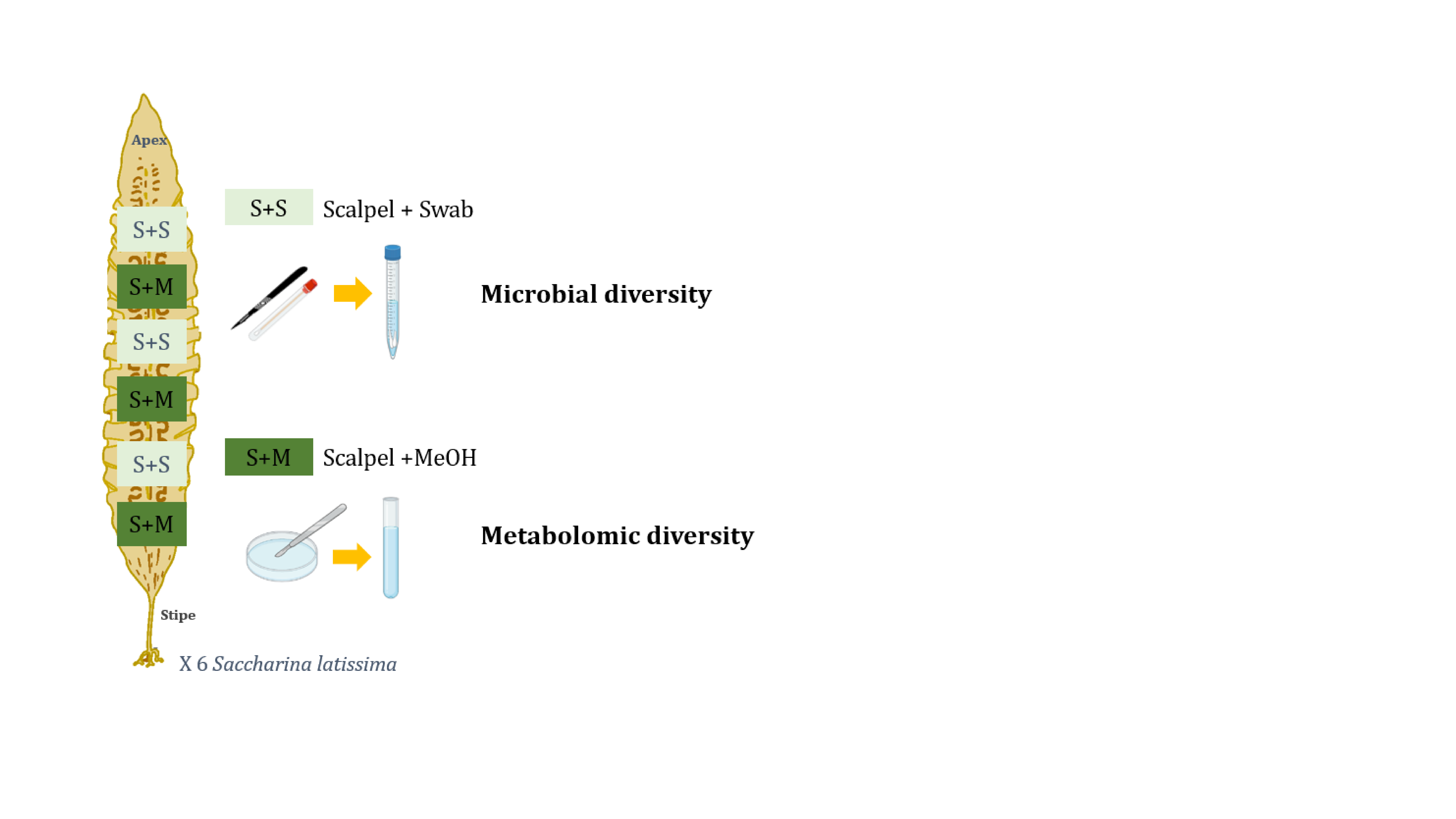

Supplement: fiae160_Supplemental_Files [file fiae160_supplemental_files.zip › Supp Fig. 1 (1).png]

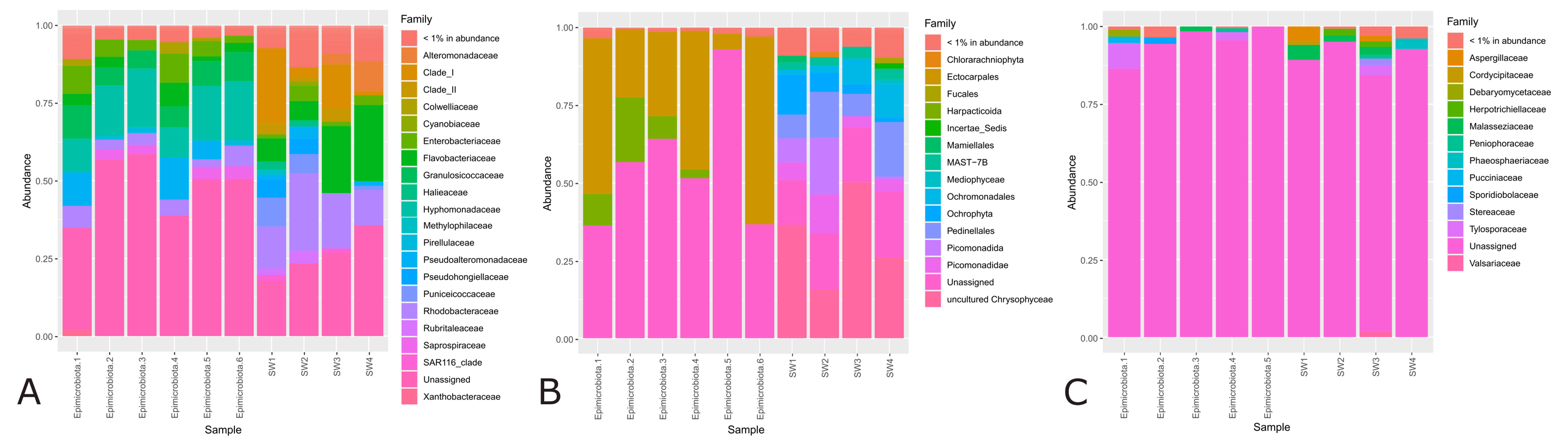

Supplement: fiae160_Supplemental_Files [file fiae160_supplemental_files.zip › Supp Fig. 2 Microbial diversity non assigned Family.png]

A

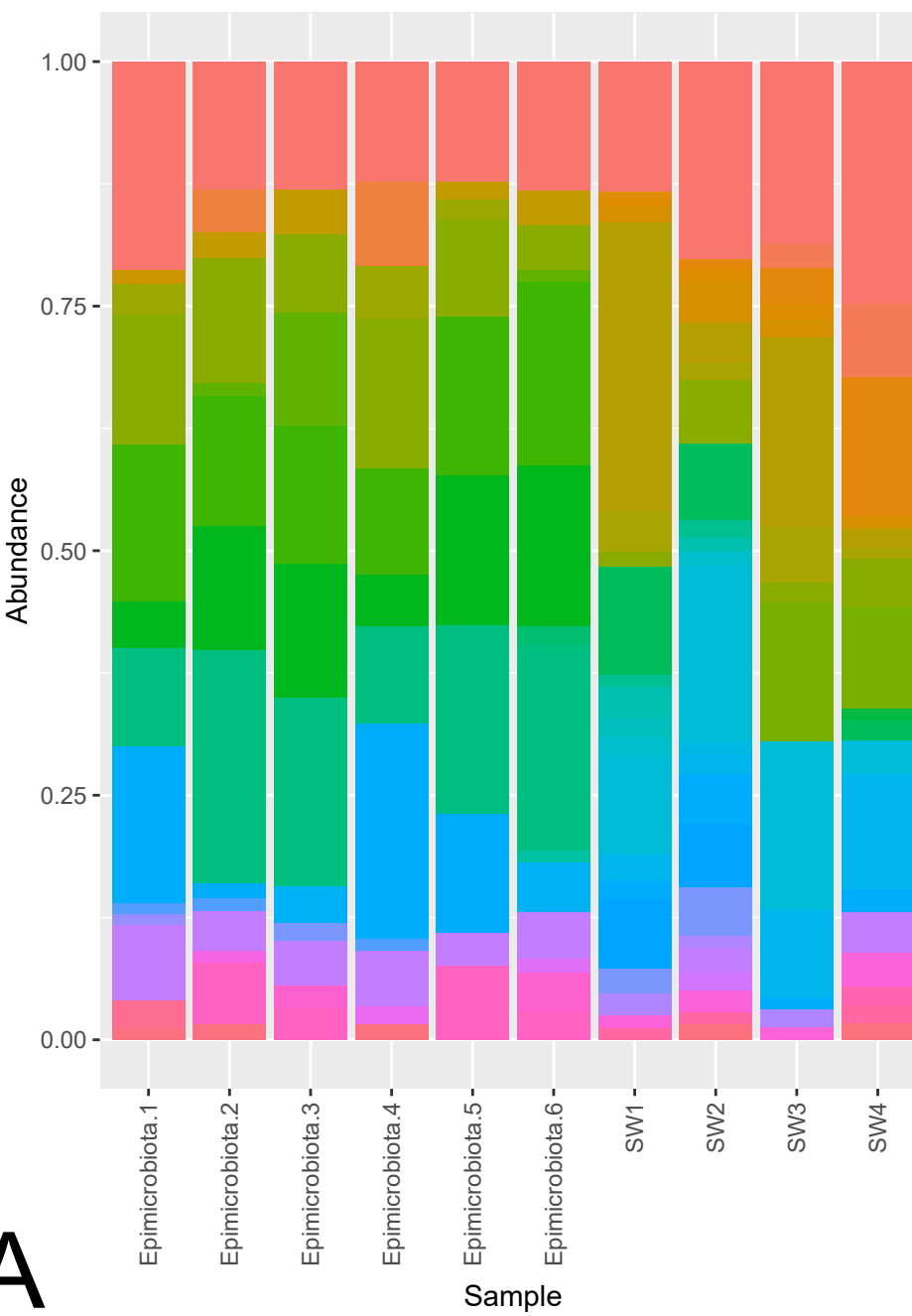

B

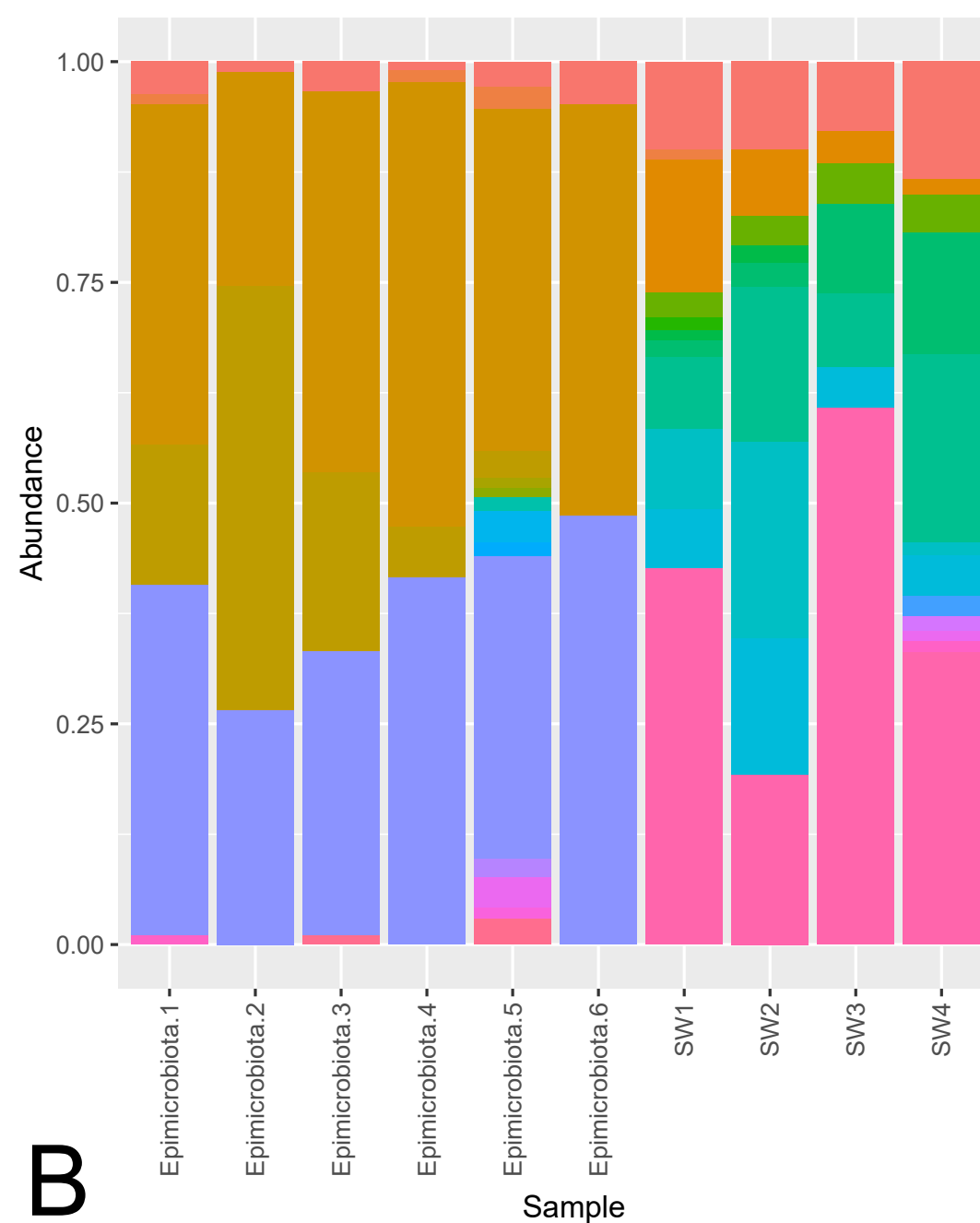

C

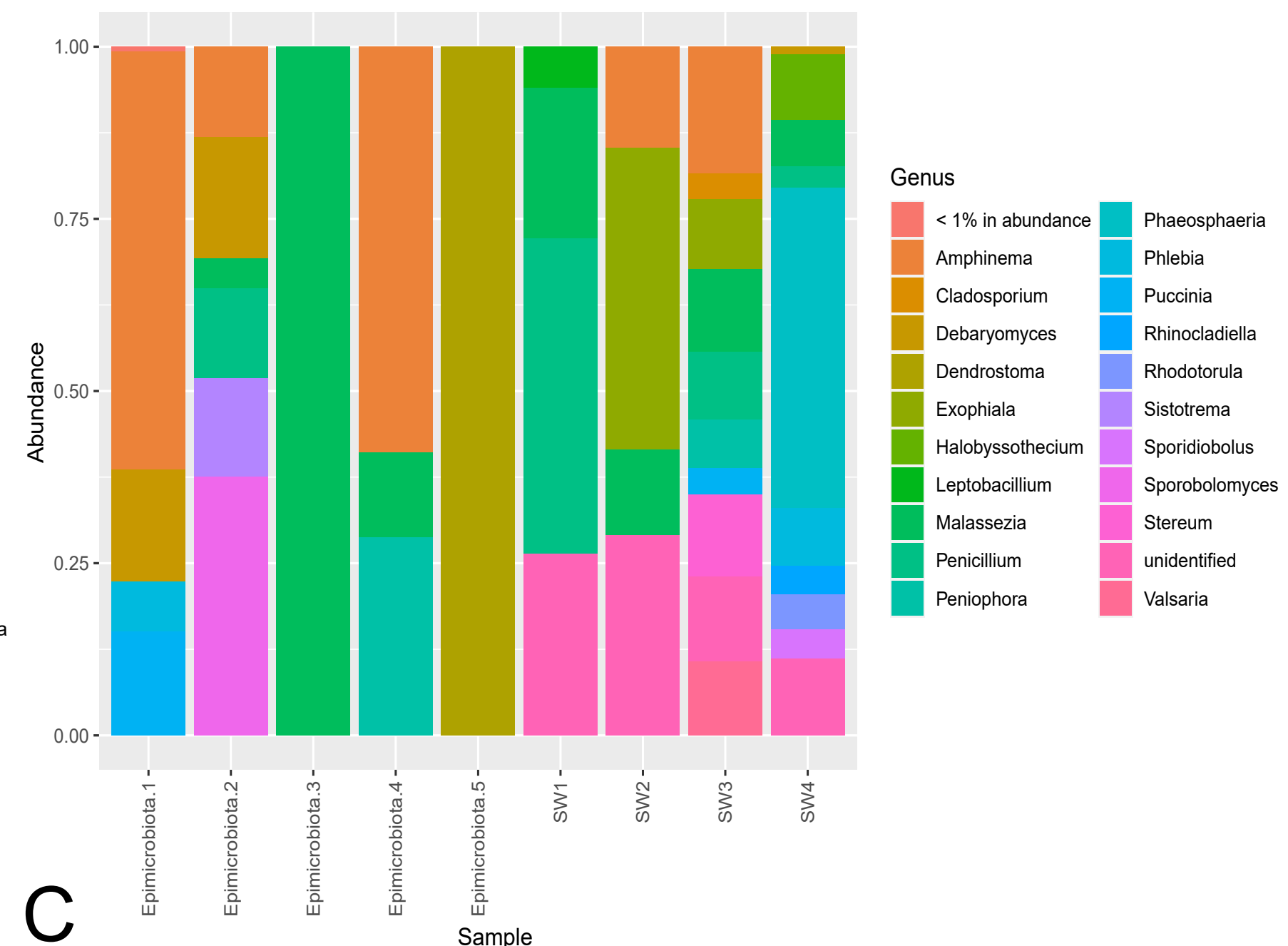

Supplement: fiae160_Supplemental_Files [file fiae160_supplemental_files.zip › Supp Fig. 3 Microbial diversity at the Genus level.pdf]

## Bacterial Diversity

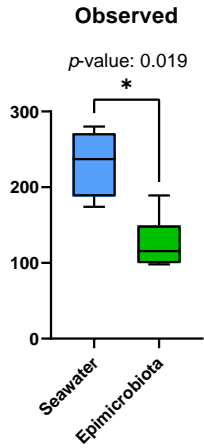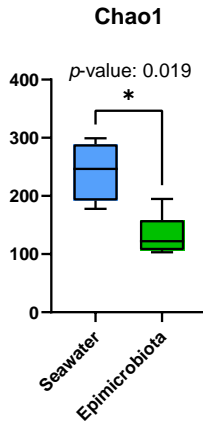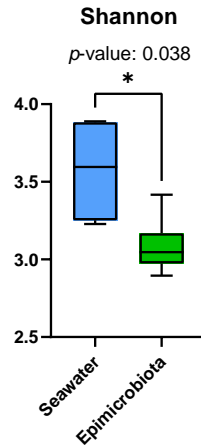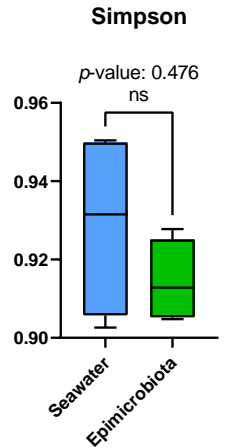

## Eukaryotic Diversity

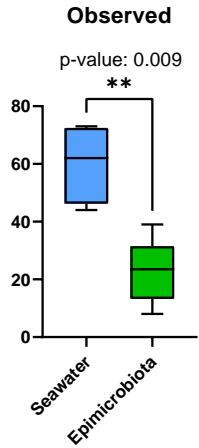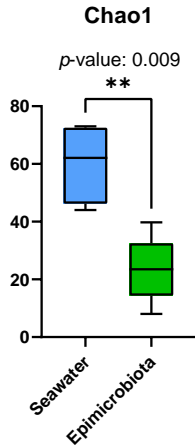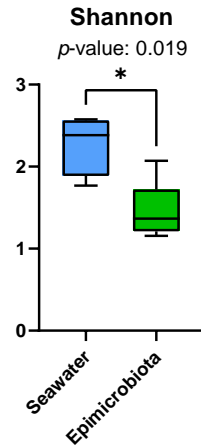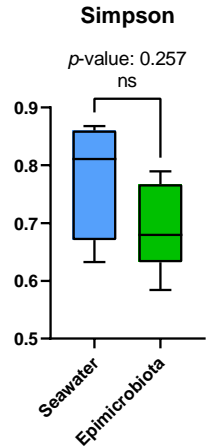

## Fungi Diversity

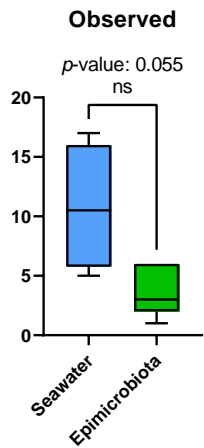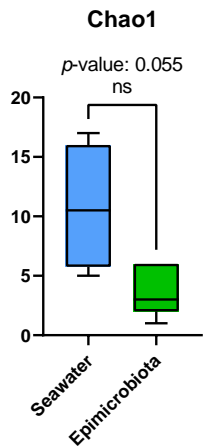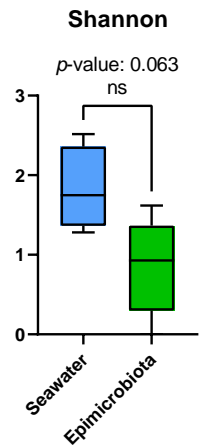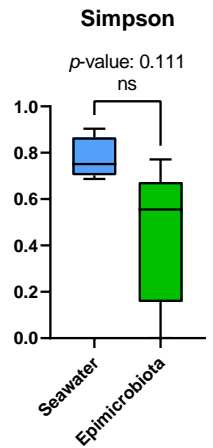

Supplement: fiae160_Supplemental_Files [file fiae160_supplemental_files.zip › Supp Fig. 4 Alpha diversity index.pdf]

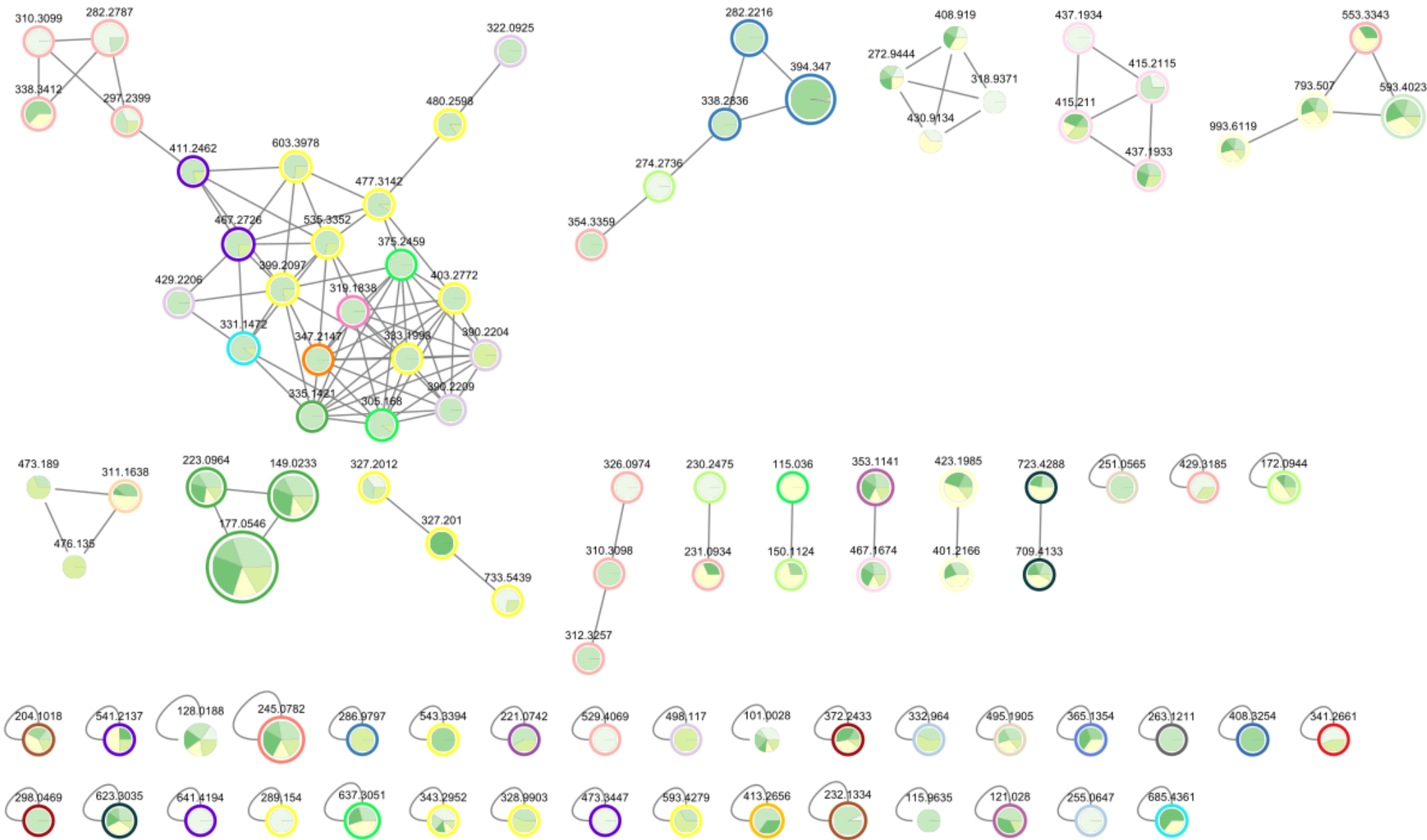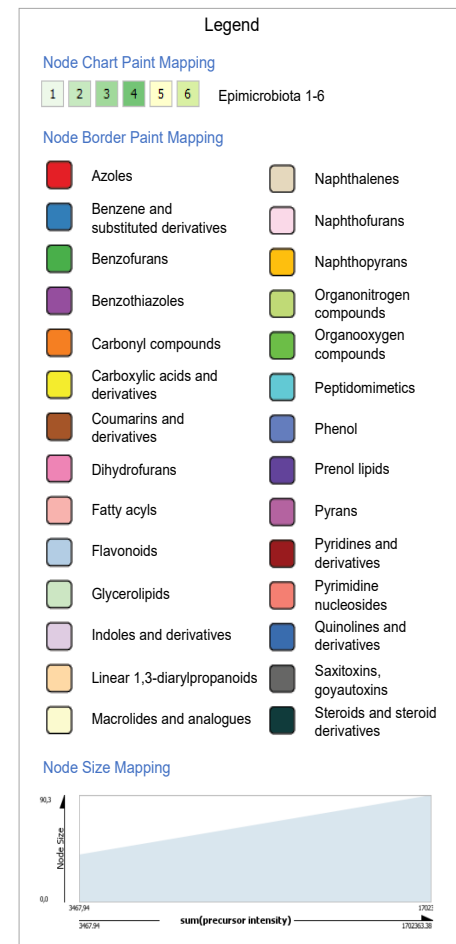

Supplement: fiae160_Supplemental_Files [file fiae160_supplemental_files.zip › Supp Fig. 5 Molecular network from FBMN without annotation.pdf]

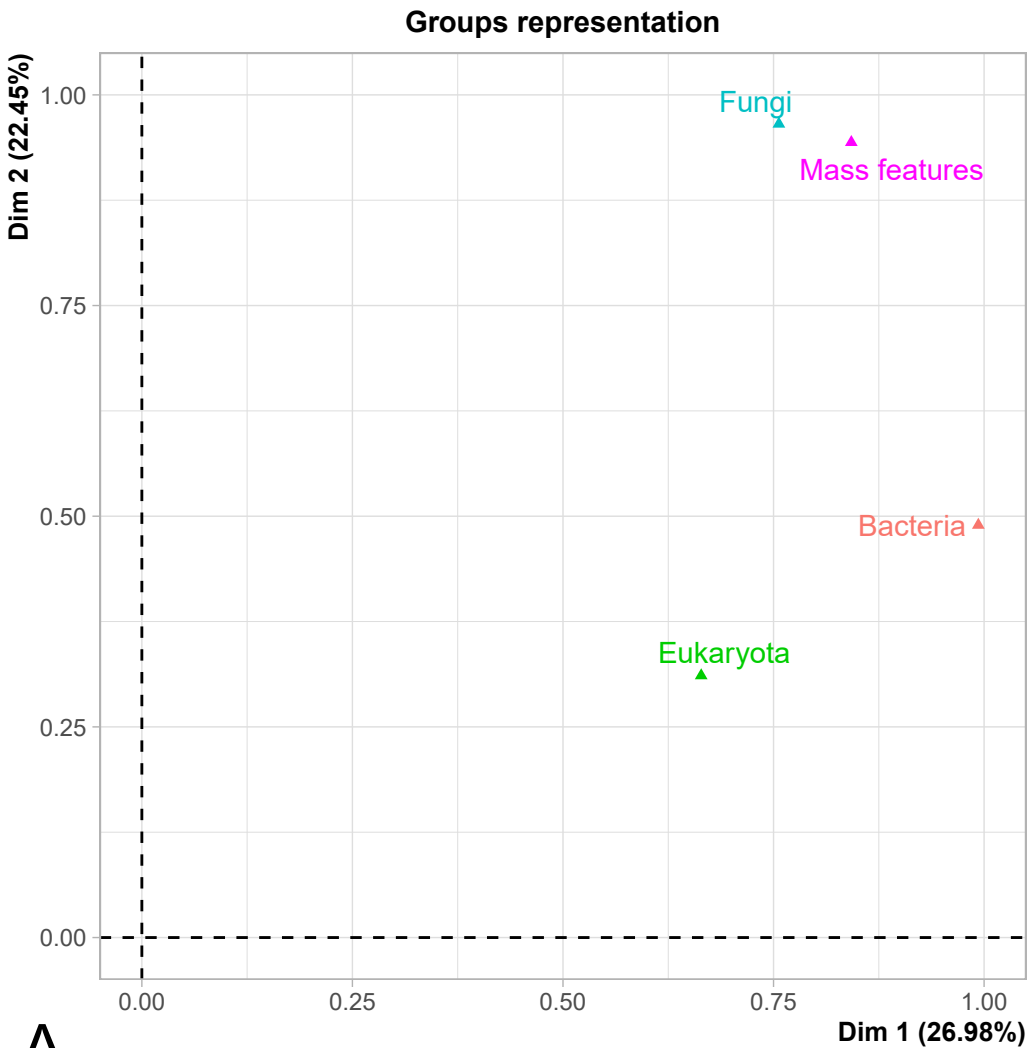**A**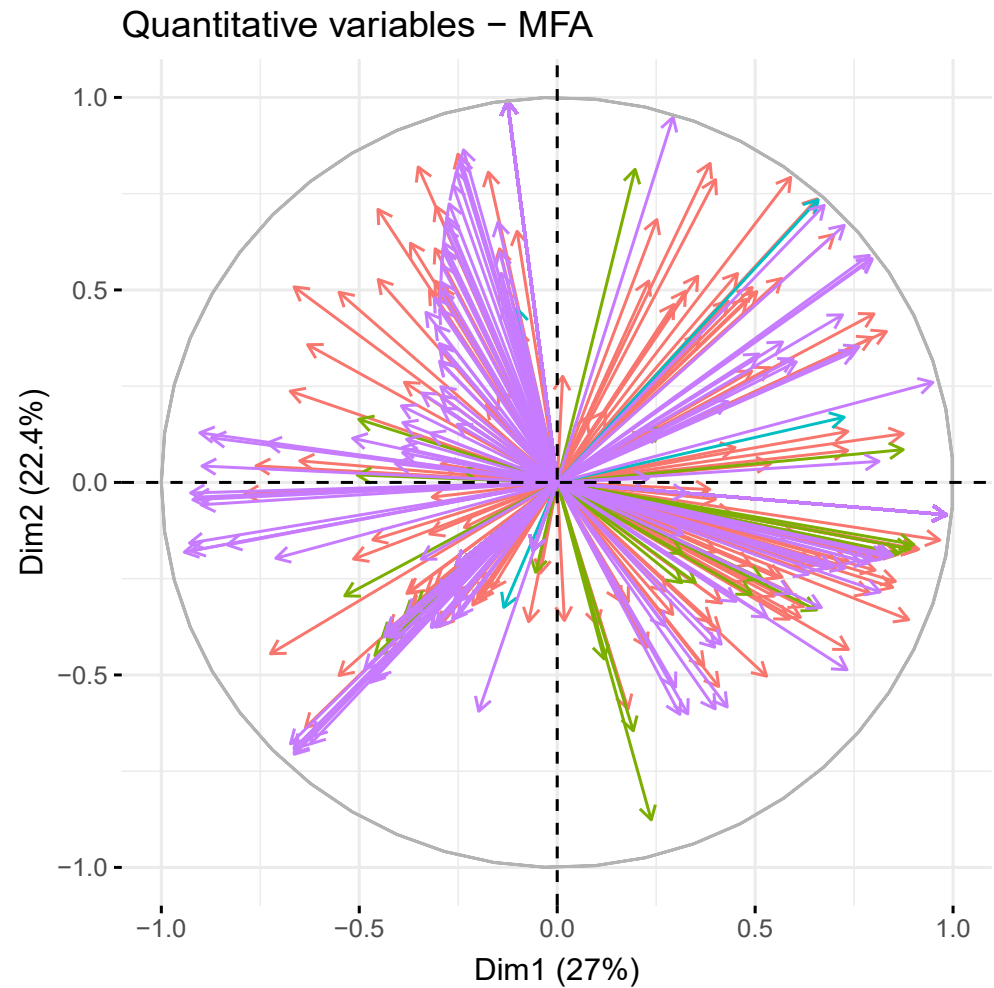**B**

Groups → Bacteria → Eukaryota → Fungi → Mass features

Supplement: fiae160_Supplemental_Files [file fiae160_supplemental_files.zip › Supp Fig. 6 MFA construction dimension.pdf]

Contribution of quantitive variables to Dim-1

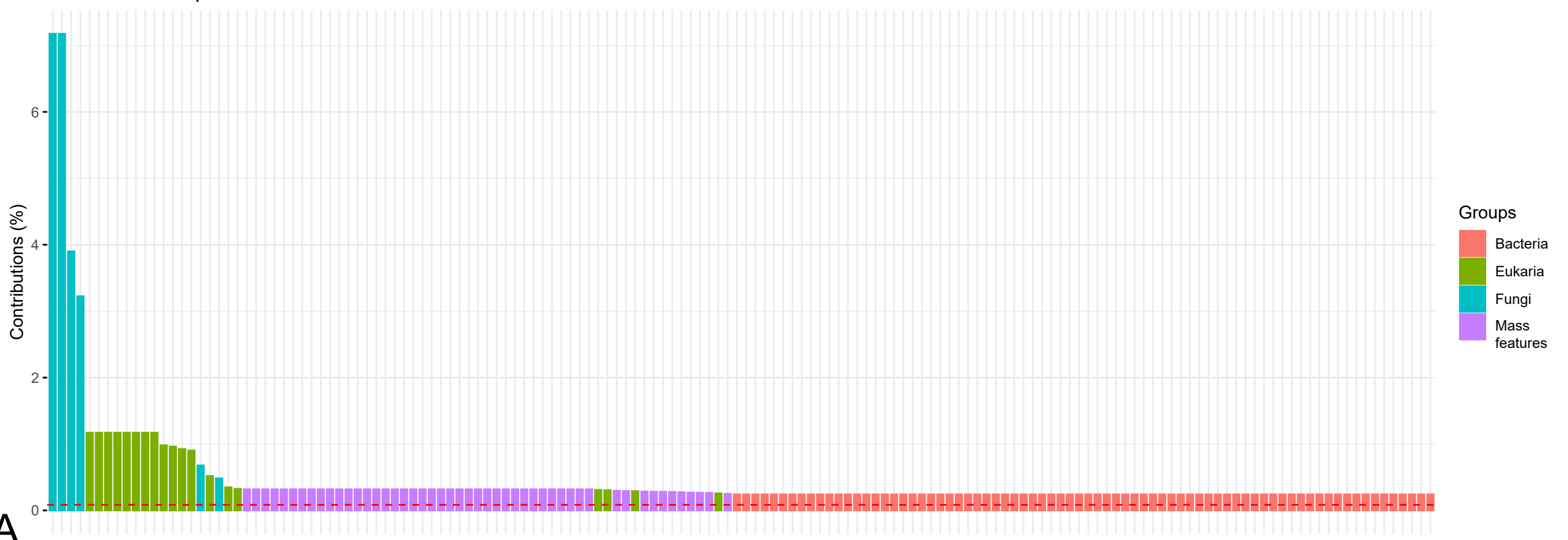

Contribution of quantitive variables to Dim-2

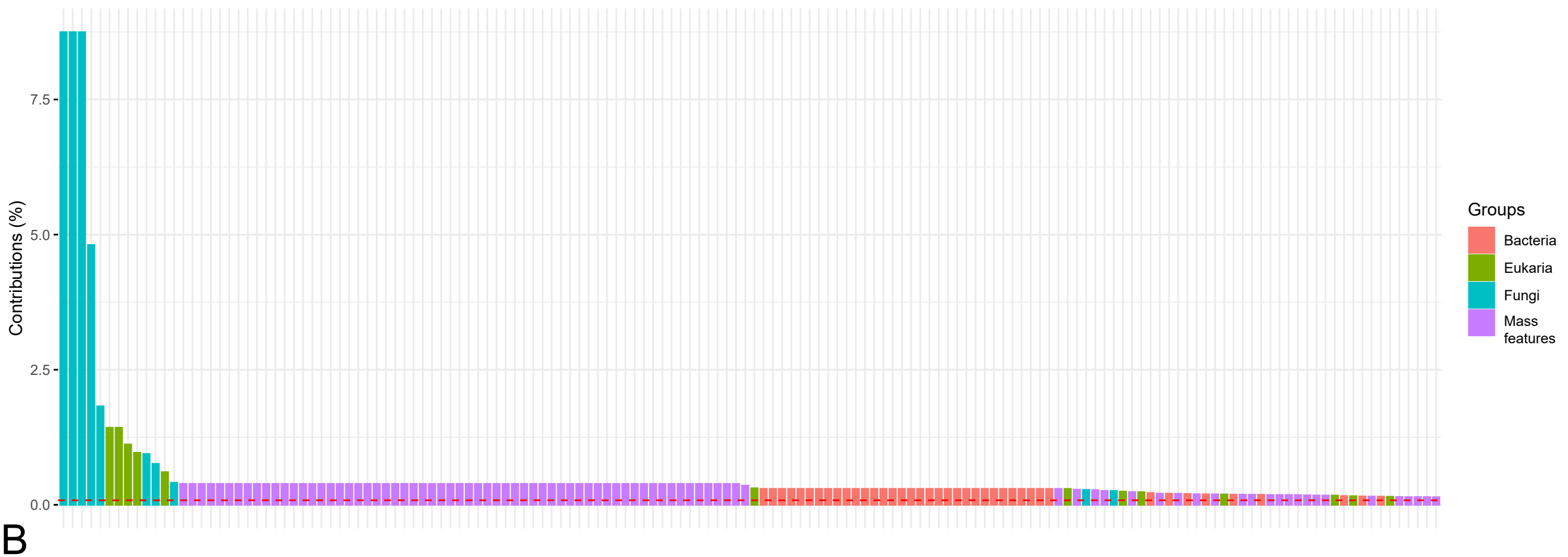

Supplement: fiae160_Supplemental_Files [file fiae160_supplemental_files.zip › Supp Fig. 7 Contribution of top 150 quantitative variables.pdf]
